# Molecular Engineering Design for High-Performance Aqueous Zinc-Organic Battery

**DOI:** 10.1007/s40820-022-01009-x

**Published:** 2023-01-13

**Authors:** Tianjiang Sun, Weijia Zhang, Qingshun Nian, Zhanliang Tao

**Affiliations:** 1 Key Laboratory of Advanced Energy Materials Chemistry (Ministry of Education), College of Chemistry, Haihe Laboratory of Sustainable Chemical Transformations, Renewable Energy Conversion and Storage Center, Nankai University, Tianjin, 300071 People’s Republic of China; 2 CAS Key Laboratory of Materials for Energy Conversion, Department of Materials Science and Engineering, Hefei National Laboratory for Physical Science at the Microscale, University of Science and Technology of China Hefei, Hefei, 230026 Anhui People’s Republic of China

**Keywords:** Aqueous Zn-organic battery, Small sulfur heterocyclic quinones, Conjugated thioether skeleton, Superlong cycling life, H^+^-involved mechanism, − 60 °C

## Abstract

Novel small sulfur heterocyclic quinones (6a,16a-dihydrobenzo[b]naphtho[2′,3′:5,6][1,4]dithiino[2,3-i]thianthrene-5,7,9,14,16,18-hexaone (4S6Q) and benzo[b]naphtho[2′,3′:5,6][1,4]dithiino[2,3-i]thianthrene-5,9,14,18-tetraone (4S4Q)) are developed by molecule structural design method and as cathode for aqueous zinc-organic batteries. The conjugated thioether (–S–) bonds as connected units not only improve the conductivity of compounds but also inhibit their dissolution by both extended π-conjugated plane and constructed flexible molecular skeleton. Hence, the Zn//4S6Q and Zn//4S4Q batteries exhibit satisfactory electrochemical performance based on 3.5 mol L^−1^ (M) Zn(ClO_4_)_2_ electrolyte. For instance, the Zn//4S6Q battery obtains 240 and 208.6 mAh g^−1^ of discharge capacity at 150 mA g^−1^ and 30 A g^−1^, respectively. The excellent rate capability is ascribed to the fast reaction kinetics. This system displays a superlong life of 20,000 cycles with no capacity fading at 3 A g^−1^. Additionally, the H^+^-storage mechanism of the 4S6Q compound is demonstrated by ex situ analyses and density functional theory calculations. Impressively, the battery can normally work at − 60 °C benefiting from the anti-freezing electrolyte and maintain a high discharge capacity of 201.7 mAh g^−1^, which is 86.2% of discharge capacity at 25 °C. The cutting-edge electrochemical performances of these novel compounds make them alternative electrode materials for Zn-organic batteries.

**Supplementary Information:**

The online version contains supplementary material available at 10.1007/s40820-022-01009-x.

## Highlights

- The conjugated thioether (–S–) bonds as connected units not only improve the conductivity of compounds but also inhibit their dissolution by both extended π-conjugated plane and constructed flexible molecular skeleton.
- The Zn//4S6Q battery based on 3.5 M Zn(ClO_4_)_2_ electrolyte shows excellent rate capacity (208.6 mAh g^−1^ at 30 A g^−1^), superlong cycling life (> 20,000 cycles with no capacity fading), and impressive low-temperature performance (201.7 mAh g^−1^ at − 60 °C).
- The H^+^-storage mechanism of 4S6Q compound is demonstrated by comprehensive characterizations.

**Supplementary Information:**

The online version contains supplementary material available at 10.1007/s40820-022-01009-x.

## Graphical abstract

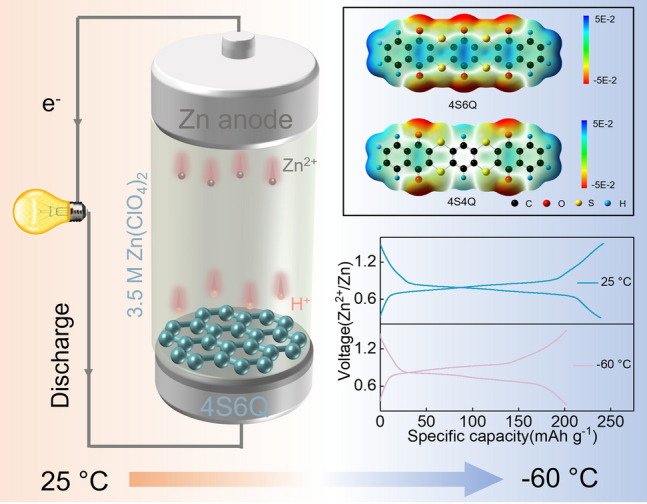

## Introduction

Developing a durable and economical energy storage device is still a challenge for collecting energy from wind, solar, tide, and so on. Compared to commercial lithium-ion batteries, aqueous zinc-ion batteries (AZIBs) possess enormous potential on large-scale energy storage devices due to many merits [1–6]. However, the energy density and cycling life of AZIBs are further limited by the lack of robust cathode materials. To solve these issues, large amounts of inorganic compounds are reported over the last several decades. For example, vanadium oxides, manganese compounds, and Prussian blue analogs are widely studied [7–9]. However, the inorganic materials have to tolerate repeating insertion/extraction of Zn^2+^. They suffer from sluggish ion diffusion kinetics and terrible structural stability due to the strong electrostatic effect between Zn^2+^ and host materials. Compared to the inorganic materials, the organic materials commonly display rapid reaction kinetics due to the rich exposed electroactive site on their surface [10–12]. Thus, that shortens the diffusion path and shows a capacitance-like behavior, which is different from inorganic materials based on ion insertion/de-insertion reaction process. Besides, the organic compounds are an alternative for AZIBs’ cathode due to many merits such as flexible structural designability, redox potential adjustability, and sustainability [13–15].

Unfortunately, the high solubility and poor conductivity of organic materials limit their development for AZIBs. For instance, calix[4]quinone (C4Q) has been reported in AZIBs and shows a high energy density [16], while its high solubility in aqueous electrolyte results in terrible cycling life. The cation-selective membrane is used to hinder the dissolution of C4Q. It cannot be ignored that the cation-selective membrane will hinder the ions transfer, which causes the unsatisfactory rate capability of C4Q. The other fatal problem of organic compounds is low conductivity, which will cause low-capacity utilization and poor rate performance [17]. For example, the pyrene-4,5,9,10-tetraone (PTO) maintains 162 mAh g^−1^ of discharge capacity at 5 A g^−1^, which is only 48.2% of capacity at 0.04 A g^−1^ (336 mAh g^−1^) [18]. Polymerization such as linear and two-dimensional polymerization (covalent organic frameworks (COF), metal–organic framework (MOF)) is a feasible strategy to solve the dissolution problem and has been widely reported. But too many inactive units are introduced in the polymer chains, which causes low specific capacity and poor conductivity [19–22].

Besides, developing novel organic compounds with low molecule weight by molecular structure design is worth considering. This strategy can allow for both conductivity and structural stability of small organic molecules by introducing heteroatoms (N, S, etc.) and extending π-conjugated plane [23–26]. In addition, the low molecule weight and the extended π-conjugated plane are in favor of improving the capacity of small organic molecules [27]. For example, the diquinoxalino [2,3-a:2′,3′-c] phenazine (HATN) and hexaazatrinaphthalene-quione (HATNQ) exhibit satisfactory electrochemical performance for AZIBs by molecule structure design [28, 29]. The introduced conjugated amine group (C=N) and extended π-conjugated plane significantly improve their conductivity and cycling stability.

Herein, considering the high theoretical specific capacity of benzoquinone (BQ) and naphthoquinone (NQ) but high dissolution and poor conductivity, the conjugated thioether (–S–) bonds are introduced to connect them (two NQ and one BQ are connected by conjugated thioether bonds, namely 4S6Q; two NQ and one benzene are connected by conjugated thioether bonds, namely 4S4Q), which can significantly improve their ion conductivity. The π-conjugated plane of whole molecules is extended, and the –S– bonds endow the molecular skeleton with high flexibility. The double effects suppress the dissolution of 4S6Q and 4S4Q materials. Additionally, rich carboxyl groups and low molecular weight endow these novel materials with high capacity. Therefore, the AZIBs based on 4S6Q cathode and 3.5 M Zn(ClO_4_)_2_ electrolyte show a high discharge capacity of 240 mAh g^−1^ and have no capacity fading after 500 cycles at 150 mA g^−1^. This system also maintains 208.6 mAh g^−1^ of discharge capacity even at a high current density of 30 A g^−1^, which is 86.9% of capacity at 150 mA g^−1^. The impressive rate capability is benefited from the pseudocapacitance behavior of ion transfer. Furthermore, the H^+^-involved reaction mechanism for 4S6Q material is confirmed with comprehensive characterizations. Excitingly, this system can stably operate at − 60 °C and obtains a high discharge capacity of 201.7 mAh g^−1^ at 150 mA g^−1^.

## Experimental and Calculation

### Preparation of 4S6Q and 4S4Q Materials

The 4S6Q compound was prepared by a simple method. 10 mmol 2, 3-dichloro-1, 4-naphthoquinone (BCNQ) was added into 100 mL deionized water, followed by adding 23 mmol hydrated sodium sulfide. The mixture was refluxed at 100 °C for two hours. Then the 5.3 mmol tetra-chloro-benzo-quinone (TCBQ) and 30 mL N, N-dimethylformamide (DMF) were added into this mixture when the temperature reduced to 70 °C and refluxed for four hours at 70 °C. The mixture was putted into 100 mL ice water and was adjusted its pH to 3 ~ 4 by 5 wt% HCl solution; then, the obtained mixture was filtrated and washed with ethanol several times. Obtained filtrate (4S6Q material) was further dry in vacuum environment overnight. The sample was further recrystallized from DMF.

The 4S4Q compound was prepared by a simple method. 10 mmol BCNQ and 23 mmol anhydrous sodium sulfide were added into 130 mL DMF solution and refluxed at 100 °C for two hours. Then the 5.3 mmol 1, 2, 4, 5-tetrachlorobenzene was added into this mixture when the temperature reduced to 70 °C and refluxed for four hours at 70 °C. The mixture was putted into 100 mL ice water and was adjusted its pH to 3 ~ 4 by 5 wt% HCl solution; then, the obtained mixture was filtrated and washed with ethanol several times. The sample (4S4Q) was further purified by recrystallizing with DMF.

### Material Characterizations

The characteristics of 4S6Q and 4S4Q powers and electrodes were conducted by Fourier transform infrared spectroscopy (FTIR, Bruker Tensor II (FTS6000)). The morphologies and microstructures of the 4S6Q and 4S4Q were tested by scanning electron microscopy (SEM, JEOLJSM-7500F) and transmission electron microscope (TEM, Talos F200X G2). Powder X-ray diffraction (XRD) was collected in the wide 2θ range of 5–50° (SmartLab 9 KW). X-ray photoelectron spectroscopy (XPS) (PerkinElmer PHI 1600 ESCA system) was used to characterize the intermediates at various charging/discharging states. All low-temperature tested were performed at ultra-low-temperature storage box (MELNG, DW-HW50). DSC was carried out in METTLER TOLEDO DSC3 in the procedure of +25~–150 °C with a cooling rate of 5 °C min^–1^, and scanned from –150 °C to +25 °C at 5 °C min^–1^.

### Electrochemical Measurements

The 4S6Q and 4S4Q electrodes are prepared by mixing as-prepared 4S6Q and 4S4Q powers, Ketjen black (KB), and polytetrafluoroethylene (PTFE) at an appropriate weight ratio of 6:3:1; the mixture is compressed into wafers (Φ 8 mm) and pressed onto stainless steel mesh (Φ 12 mm). Then the electrodes are dried at 80 °C for 12 h under vacuum. The mass loading of 4S6Q and 4S4Q compounds was 1–2 mg cm^−2^. The 2032-type coin cells are assembled by 4S6Q or 4S4Q cathode, 3.5 M Zn(ClO_4_)_2_ electrolyte, Zn metal (0.05 mm, Φ 12 mm) anode, and glass fiber separator (Φ 16 mm). CV tests were carried out on an electrochemical workstation (CHI660E). The galvanostatic charge/discharge tests were implemented after resting 5 h by using Neware battery test system (CT-4008, Shenzhen, China). The tested voltage range of Zn//4S6Q was 0.3 ~ 1.5 V versus Zn^2+^/Zn, and the tested voltage range of Zn//4S4Q was 0.3 ~ 1.2 V vs. Zn^2+^/Zn. The current density and specific capacity of full battery were based on the active mass of cathode.

### Computational Details

Density functional theory (DFT) calculations are performed by using the Gaussian 16 program[30]. Geometry optimization and frequency analyses are performed in water solvent with the SMD solvation model. C, H, O, S are using B3LYP functional and 6–31 + G (d, p) basis set. The calculated results of HOMA and LOL-π were performed with Multiwfn 3.8 programs [31].

To distinguish the type of charge transfer kinetics, the *b* values can be fitted by Eq. (1) [10]:

1 $$i = av^{b}$$

where the *i* is peak current (mA), and the *v* is scan rate (mV s^−1^). The *b* values can be fitted by the linear relation of log(*i*) = *b*log(*v*) + log(*a*).

The ratios of capacitance contribution and diffusion contribution are calculated following Eqs. (2) and (3) [32]:

2 $$i = k_{1} v + k_{2} v^{\frac{1}{2}}$$

3 $$\frac{i}{{v^{\frac{1}{2}} }} = k_{1} v^{\frac{1}{2}} + k_{2}$$

where $k_{1} v$ refers to the current part of capacitance contribution and $k_{2} v^{\frac{1}{2}}$ refers to the current part of diffusion contribution.

The ions diffusion coefficient (*D*) is calculated by galvanostatic intermittent titration technique (GITT) measurement, which follows Eq. (4) [10]:

4 $$D = \frac{{4L^{2} }}{\pi \tau }\left( {\frac{{\Delta E_{s} }}{{\Delta E_{t} }}} \right)^{2}$$

where τ is the relaxation time. Δ*E*_s_ is the steady-state voltage change after current pulse. Δ*E*_t_ is the voltage change (*V*) during the relaxation process. *L* is ion diffusion length (cm), which is approximately equal to the thickness of electrode.

## Results and Discussion

### Synthesis and Characterizations

**Fig. 1:**
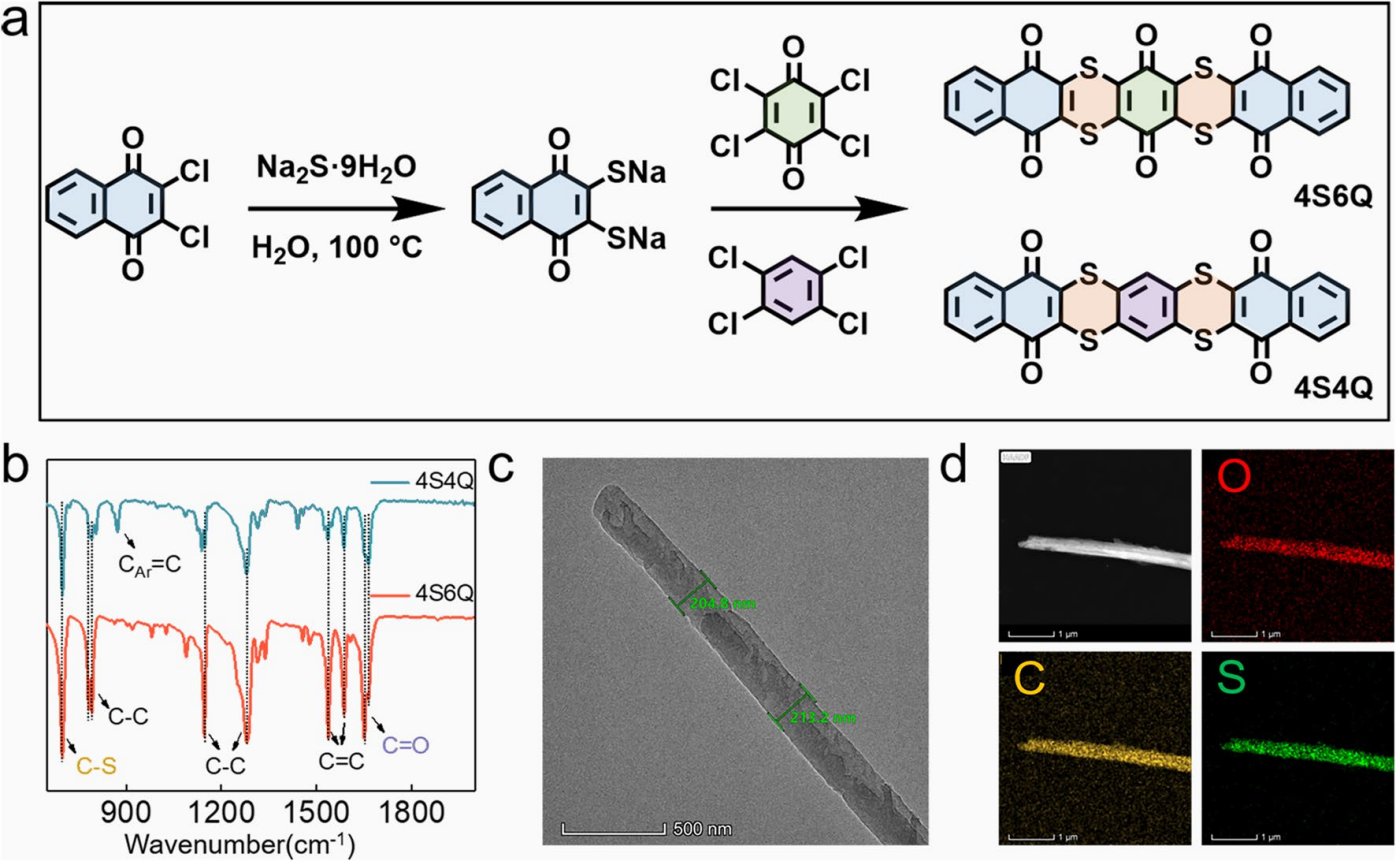
Synthesis and characterizations. **a** The synthesis processes of 4S4Q and 4S6Q compounds. **b** FTIR spectra of 4S4Q and 4S6Q compounds. **c** TEM image of 4S6Q sample. **d** The TEM-mapping images of 4S6Q sample

The NQ and BQ show high theoretical specific capacity but low-capacity utilization due to limited conjugated plane and poor conductivity (Fig. S1). Moreover, these small organic materials face a deadly problem of dissolution, resulting in poor cycling life. Thus, the conjugated thioether (–S–) bonds are considered to link the NQ and BQ units. Firstly, the introduction of –S– bonds can improve the conductivity of compounds due to two unpaired electrons on the S element. Secondly, the extended π-conjugated plane with conjugated thioether skeleton can raise the structure stability of compounds and suppress their dissolution. Finally, the compounds with conjugated thioether skeleton exhibit a characteristic of combining rigidity and softness because of the rigid benzene ring and flexible thioether bond, which show a high tolerance to allow reversible de-/insertion of ions [33–36]. Inspired by the above points, the compounds that –S– bonds connect two NQ molecules and one BQ molecule (namely 4S6Q (6a,16a-dihydrobenzo[b]naphtho[2′,3′:5,6][1,4]dithiino[2,3-i]thianthrene-5,7,9,14,16,18-hexaone)) or one benzene (namely 4S4Q (benzo[b]naphtho[2′,3′:5,6][1,4]dithiino[2,3-i]thianthrene-5,9,14,18-tetraone)) are successfully synthesized by simple two-step reactions (Fig. 1a). The Fourier transform infrared (FTIR) spectra in Fig. 1b confirm their structure. The stretching vibration peaks at 1650 and 1663 cm^−1^ refer to the signal of C=O groups. And the peak at 700 cm^−1^ corresponds to the groups of C–S [33, 35]. Noted that a peak at 872 cm^−1^ is only detected in as-prepared 4S4Q, which can be attributed to the stretching vibration of benzene. The ^1^H nuclear magnetic resonance (^1^H NMR) spectra are collected and shown in Fig. S2 and S3. No other H signals are detected on the ^1^H NMR spectra, indicating that the as-synthesis samples are pure. The 4S6Q and 4S4Q show a rod-like morphology by scanning electron microscopy (SEM) images (Figs. S4 and S5). However, the 4S4Q material shows more serious agglomeration than the 4S6Q material, which may influence its electrochemical performance. Further, the width of the rod for 4S6Q is about 210 nm by the transmission electron microscopy (TEM) tests, corresponding to a short diffusion path of ions (Fig. 1c). And from the TEM-mapping images in Fig. 1d, the C, O, and S elements are evenly distributed in the rod. The TEM and TEM mapping images of 4S4Q are shown in Figs. S6 and S7. The crystal characteristics of these compounds are collected by X-ray diffraction (XRD). As shown in Fig. S8, the 4S6Q displays better crystallinity than 4S4Q, which may relate to the different molecular arrangements.

### Density Functional Theory Calculations

**Fig. 2:**
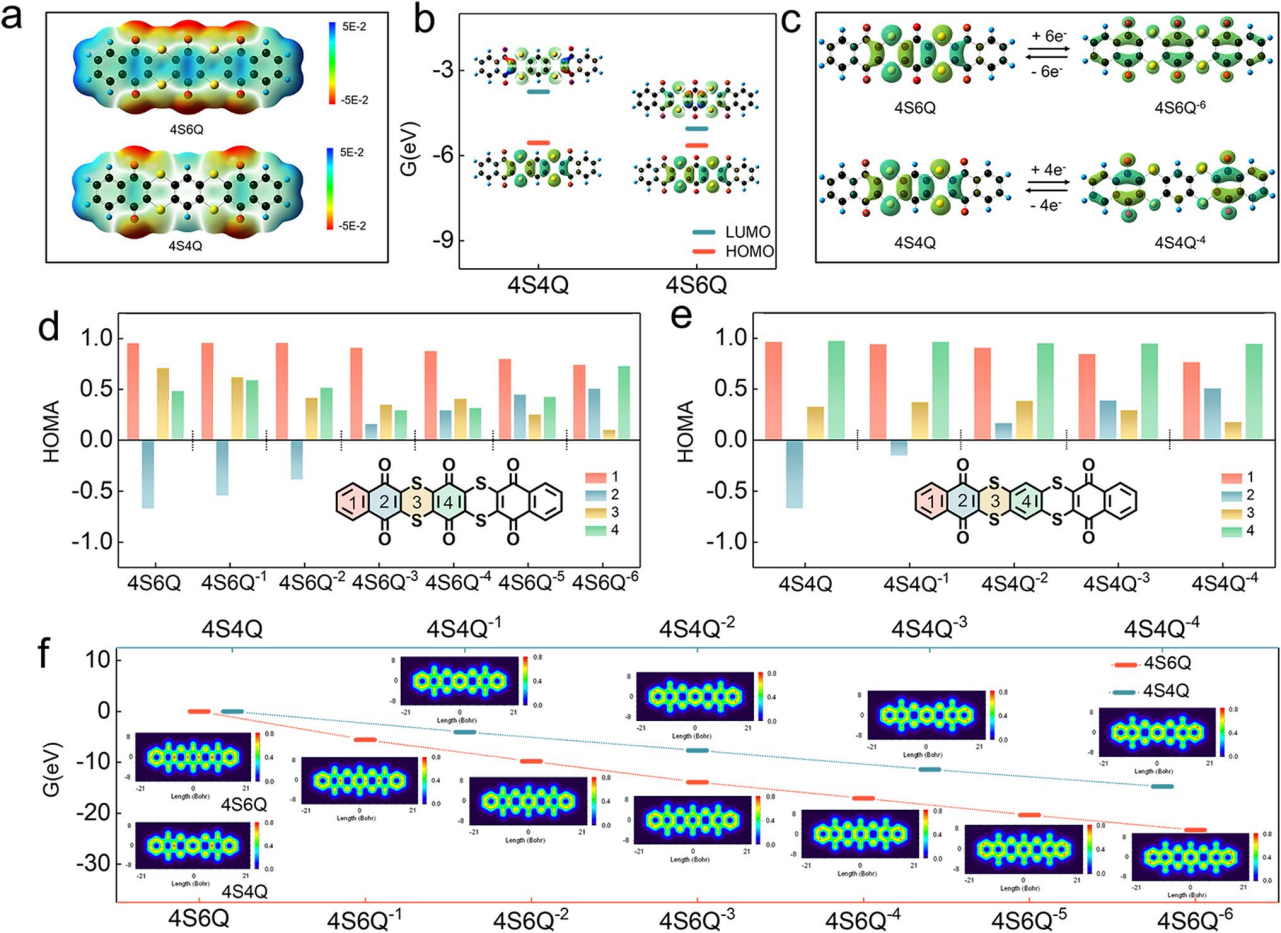
DFT calculations. **a** The MESP image of 4S4Q and 4S6Q molecules. **b** Calculated LUMO and HOMO plots of 4S4Q and 4S6Q molecules. **c** Calculated HOMO plots of 4S4Q, 4S4Q^4−^, 4S6Q, and 4S6Q.^6−^ molecules. **d** Calculated HOMA values of 4S6Q and corresponded reduction states. **e** Calculated HOMA values of 4S4Q and corresponded reduction states. **f** Energy level of neutral and reduced 4S6Q and 4S4Q molecules (the inserted images reflect the simulative LOL-π)

The electrochemical activities of 4S4Q and 4S6Q are studied by DFT calculations. Firstly, the optimized planar structures of 4S4Q and 4S6Q are obtained (Fig. S9). The electrostatic potential (ESP) can predict the reaction site of organic molecules. As shown in Fig. 2a, the carbonyl groups (C=O) for the two compounds all show negative ESP, indicating that they tend to occur in electrophilic reactions [16, 28]. The cations will bind with the negative charge center (C=O) during the discharged process. Thus, the carbonyl groups are regarded as the reaction active sites to store cations. Moreover, the more carbonyl groups for 4S6Q imply a higher theoretical specific capacity (295 mAh g^−1^) than the 4S4Q compound (209 mAh g^−1^). The highest occupied molecular orbital (HOMO) and the lowest unoccupied molecular orbital (LUMO) are calculated and shown in Fig. S10 and 2b. The energy gaps between LUMO and HOMO for 4S4Q and 4S6Q are smaller than other organic monomers, reflecting a good ability for electron transfer [25, 27, 37]. That is to say, introducing –S– bonds to an organic skeleton can significantly raise its electronic conductivity. The 4S6Q shows a lower energy gap of 0.60 eV than 4S4Q (1.81 eV), which is benefited from the better π-conjugated effect (Fig. 2b). The electric conductivities of 4S4Q and 4S6Q compound are further tested. As shown in Fig. S11, the 4S4Q and 4S6Q show high electric conductivity of 8.87 and 9.45 S cm^−1^, which are in agreement well with the DFT calculations. Additionally, the 4S6Q displays a lower energy level of LUMO, indicating that 4S6Q has a higher electron affinity and higher reduction potential [25, 38]. The HOMO plots of organic molecules can provide information on the ability to achieve maximum capacity [39]. As shown in Fig. 2c, effective electron delocalization occurs in the conjugated structure of reduced 4S6Q (4S6Q^6−^) when 4S6Q molecular accepted six electrons, suggesting all carbonyl groups can be reduced in theory. Similarly, the 4S4Q molecular has the ability to accept four electrons. The π electron on the organic skeleton reflects its π-conjugated effect, which can further evaluate its structural stability. Thus, the localized orbital locator-π (LOL-π) method is employed to reveal the π-electron delocalization behavior [40, 41]. As shown in Fig. S12, the π electrons for NQ and BQ molecules are well delocalized in the C=C and C=O skeletons. When they are connected by –S–, the delocalization paths have not been changed, and the π-conjugated planes are efficiently expanded, which ensures their good structural stability. Besides the LOL-π method, aromaticity can also evaluate the structural stability of organic materials [42]. According to Hückel’s rule, the neutral 4S6Q and 4S4Q with 4n (*n* = 1, 2, 3, etc.) π electrons show antiaromaticity [43]. Although the anti-aromatic molecules show higher energy and lower stability than aromatic molecules, they display excellent charge transport properties and high redox activity [44]. Here, the harmonic oscillator model of aromaticity (HOMA) index is used to evaluate the aromaticity of each ring for 4S6Q and 4S4Q molecules at different reduced states [45]. As shown in Fig. 2d, e, the HOMA indexes of ring 2 for the neutral 4S6Q and 4S4Q are negative, corresponding to an anti-aromatic character, and the other rings show aromatic character. When they are progressively reduced (accept electrons), each ring shows positive HOMA index, suggesting improved aromaticity for whole molecule. Commonly, the quinone derivatives are easily dissolved in electrolyte when they are reduced. From the results of HOMA indexes, the stability of 4S6Q and 4S4Q compounds is improved when they are reduced, implying that they have good cycling stability. The LOL-π mapping images of 4S6Q and 4S4Q compounds at different reduced states are calculated and shown in Fig. 2f. The *π* electrons can be well delocalized in the framework of 4S6Q and 4S4Q compounds after accepted electrons, especially in quinone rings, indicating improved aromaticity and stability. The energies of 4S6Q and 4S4Q have gradually decreased when progressively accepted electrons, indicating the efficient utilization of the C=O groups.

### Electrochemical Performance

**Fig. 3:**
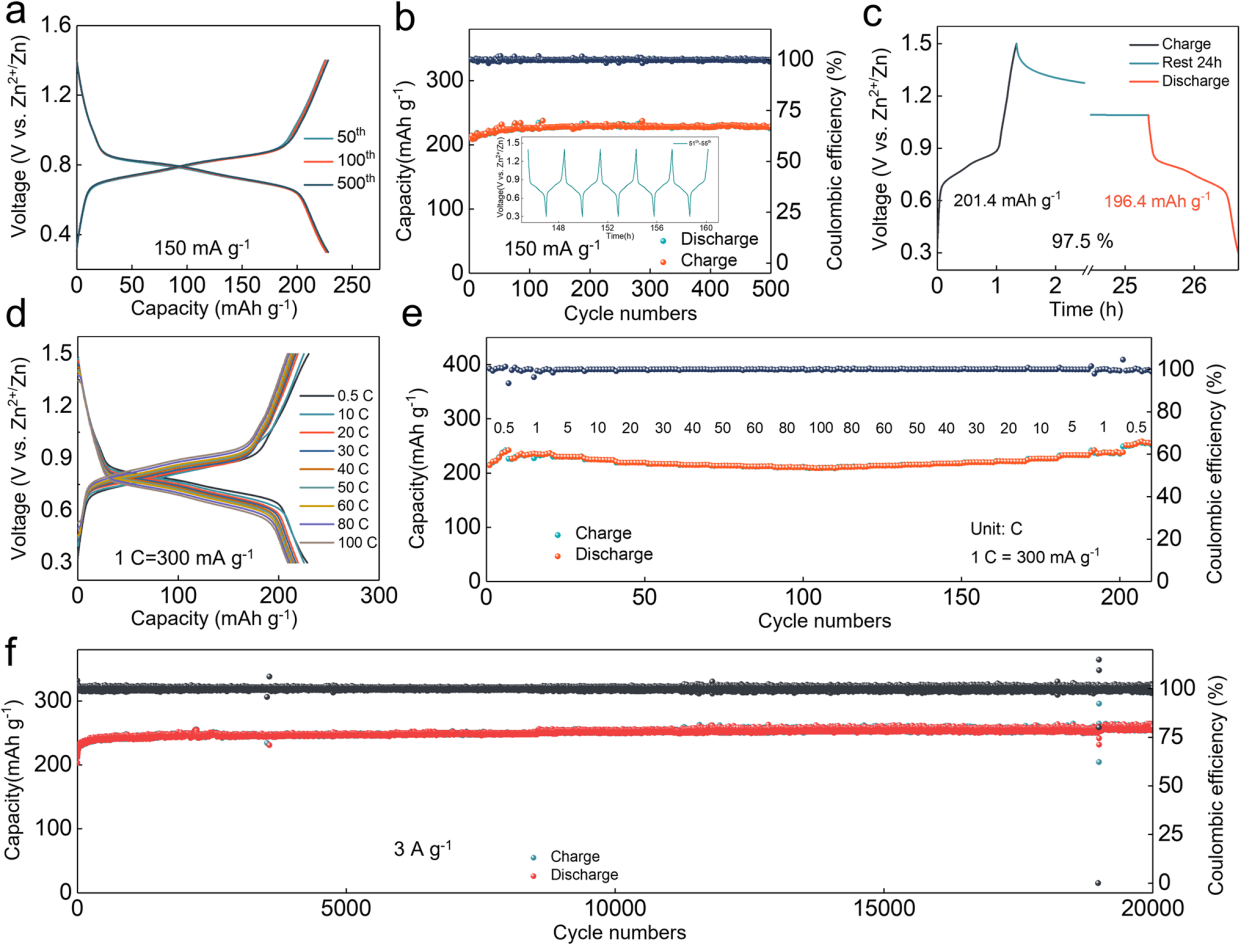
Electrochemical performance. **a** GCD curves of Zn//4S6Q battery at 150 mA g^−1^. **b** Cycling stability of Zn//4S6Q battery at 150 mA g^−1^. **c** Self-discharge performance of Zn//4S6Q battery. **d** GCD curves of Zn//4S6Q battery at different current densities. **e** Rate performance of Zn//4S6Q battery. **f** Cycling stability of Zn//4S6Q battery at 3 A g.^−1^

The AZIBs are fabricated based on the 3.5 M Zn(ClO_4_)_2_ electrolyte to verify the electrochemical feasibility of 4S6Q and 4S4Q compounds. The 3.5 M Zn(ClO_4_)_2_ electrolyte shows an ultralow freezing point of − 118 °C (Fig. S13), which can give AZIBs a potential low-temperature electrochemical performance. The cyclic voltammetry (CV) tests of Zn//4S6Q and Zn//4S4Q batteries are firstly performed (Fig. S14). The Zn//4S6Q and Zn//4S4Q batteries also show a pair of obvious redox peaks, and the shape and intensity of redox peaks are consistent after 10th cycles, indicating that 4S6Q and 4S4Q compounds possess excellent electrochemical reversibility. Note that, the 4S6Q shows higher reduction potential and smaller voltage polarization than 4S4Q, which may be benefited from its low energy level of LUMO and small energy gap between LUMO and HOMO. These results also are reflected in the galvanostatic charge/discharge (GCD) curves. As shown in Fig. S15, the discharge platform voltage of 4S6Q is higher than that of 4S4Q. The Zn//4S6Q battery obtains a high discharge capacity of 240 mAh g^−1^ at 150 mA g^−1^ (81.4% of theoretical specific capacity), while the discharge capacity of the Zn//4S4Q battery only has 145 mAh g^−1^ (69.4% of theoretical specific capacity), which can be attributed to the serious agglomeration behavior of the as-prepared 4S4Q. Significantly, the Zn//4S6Q battery can achieve a high discharge capacity of 273 mAh g^−1^ at a current density of 60 mA g^−1^ (Fig. S16). As shown in Fig. 3a, the voltage polarization of Zn//4S6Q battery has not increased after 500 cycles. Meanwhile, this system has not capacity fading over 500 cycles at a low current density of 150 mA g^−1^, and it maintains high Coulombic efficiency near 100% and stable voltage platform during the cycling process (Fig. 3b). The self-discharge performance of 4S6Q is tested to evaluate its durability. The battery is firstly charged to 1.5 V and then is discharged to 0.3 V after resting for 24 h. As shown in Fig. 3c, the Coulombic efficiency can maintain 97.5%, reflecting a low self-discharge behavior for 4S6Q. Additionally, the 4S6Q displays an impressive rate capability due to its high electronic conductivity. The Zn//4S6Q battery still maintains a high discharge capacity of 208.6 mAh g^−1^ even at an ultrahigh current density of 30 A g^−1^, which is 91.4% of discharge capacity at 150 mA g^−1^ (Fig. 3d). The voltage polarization of this system only slightly increased. The discharge capacity still can recover to the initial level when the current density returns to 150 mA g^−1^, indicating good reversibility of 4S6Q (Fig. 3e). The cycling stability of Zn//4S6Q battery is tested at 1 and 3 A g^−1^ and has no capacity reduction after 5000 and 20,000 cycles, respectively (Figs. S17 and 3f), which are superior to reported works (Table S1). It is worth noting that the voltage polarization has gradually decreased and then remains stable during the cycling process (Fig. S18). The significant cycling stability is benefited from the extended π-conjugated plane and limited solubility. Inspired by the excellent electrochemical performance of 4S6Q, the pouch cell is fabricated to verify its potential application. As shown in Fig. S19, two pouch cells can all normally operate when they are connected in series and parallel, respectively. The electrochemical performance of Zn//4S4Q batteries is also collected and showed satisfactory rate capability and cycling stability (Figs. S20 and S21).

### Studies of Reaction Kinetics

**Fig. 4:**
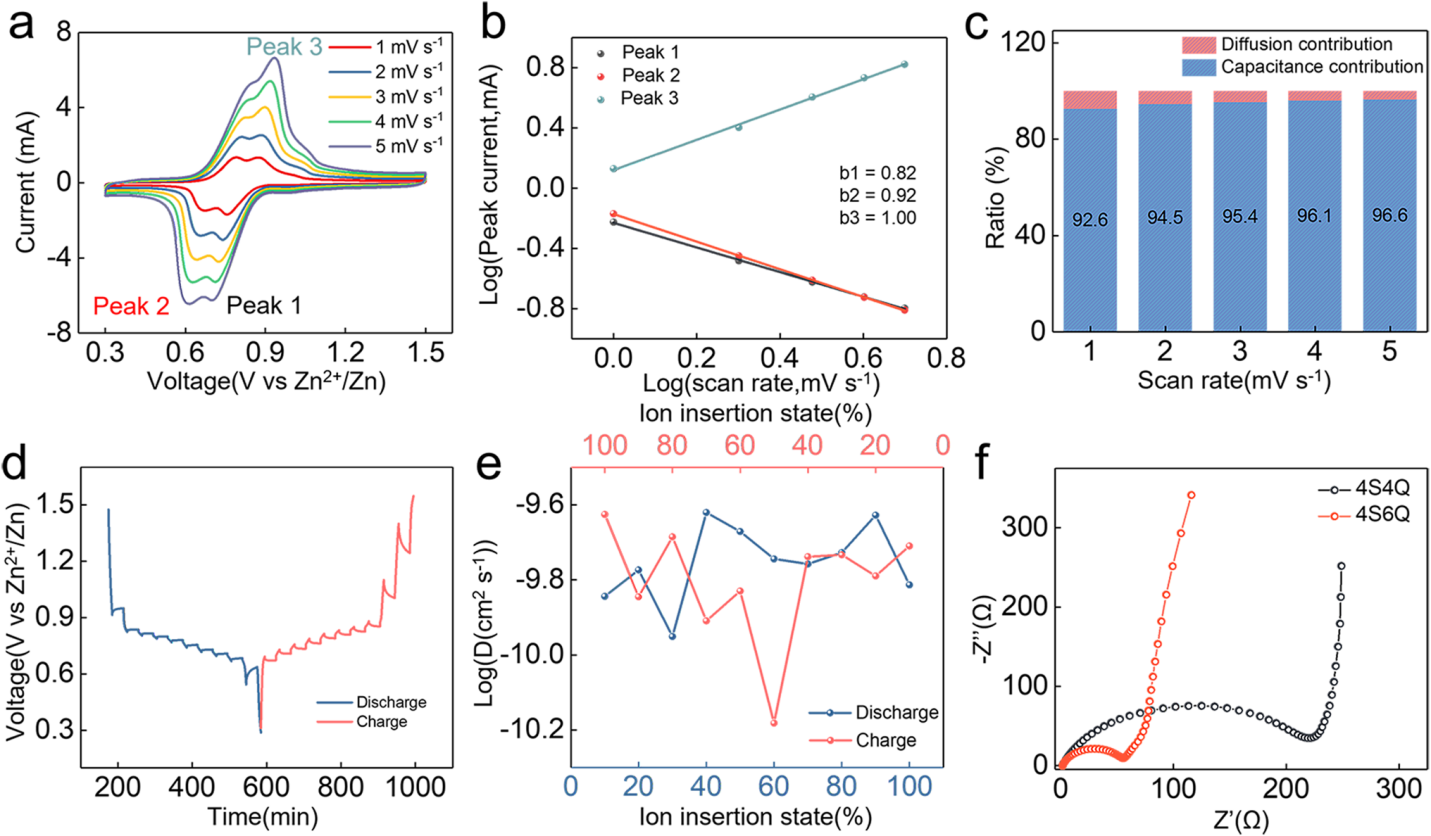
The studies of reaction kinetics. **a** CV curves of Zn//4S6Q battery at different scan rates. **b** The calculated *b* values for different peaks. **c** Calculated capacitance/contribution ratios at different scan rates. **d** Charge–discharge curves of Zn//4S6Q battery for GITT test. **e** Calculated D_*ions*_ by GITT test, f the EIS of 4S4Q and 4S6Q

The CV curves of Zn//4S6Q battery at different scan rates are performed to distinguish the charge storage types (Fig. 4a). According to the linear relationship between log(*i*, peak current (mA)) and log(*v*, scan rate (mV s^−1^)), the *b* values of peak 1, peak 2, and peak 3 are fitted to be 0.82, 0.92, and 1.00, suggesting that the charge storage behavior is dominated by both capacitance and diffusion control (Fig. 4b) [46]. The capacitance behavior commonly shows a fast charge transfer kinetics, rendering a good rate performance for batteries. Thus, the ratios of diffusion contribution and capacitance contribution are further calculated. As shown in Fig. 4c, the ratio of capacitance contribution exceeds 92.6% at different scan rates, indicating that the 4S6Q has fast reaction kinetics. The ions diffusion coefficient (*D*_ions_) is revealed by galvanostatic intermittent titration technique (GITT) measurement (Fig. 4d). As shown in Fig. 4e, the calculated *D*_ions_ values are as high as 10^−10^ ~ 10^−9^ cm^2^ s^−1^, which is benefited from the high conductivity of 4S6Q compound. The high capacitance/contribution ratio and high *D*_ions_ values give the 4S6Q an excellent rate capability. The same studies about charge storage types are carried out on Zn//4S4Q battery. The *b* values are calculated to be 0.85 and 0.91 for peak 1 and peak 2 by the CV curves of different scan rates (Figs. S22a, b), corresponding to a pseudocapacitance behavior. The capacitance/contribution ratios raise from 89.6 to 95% with increased scan rates (Fig. S22c). These results suggest that introduced –S– bonds can improve the conductivity of the whole molecule and facilitate charge transfer. Moreover, electrochemical impedance spectroscopy (EIS) is conducted. To avoid Zn anode effect, the 4S6Q or 4S4Q electrode is firstly reduced and then as an anode to fabricate the battery. As shown in Fig. 4f, the semicircle of 4S4Q_reduced_//4S4Q battery in the high-frequency region is larger than that of 4S6Q_reduced_//4S6Q battery, revealing that the charge transfer resistance (*R*_ct_) of 4S4Q_reduced_//4S4Q battery is much higher than that of 4S6Q_reduced_//4S6Q battery. According to the linear relationship between ionic conductivity (σ) and 1/*R*_ct_, the calculated ionic conductivities of 4S4Q and 4S6Q are 4.78 and 11.76 mS cm^−1^, respectively (Fig. S23). Thus, the low *R*_ct_ of 4S6Q_reduced_//4S6Q battery can be assigned to the high conductivity of the 4S6Q compound.

### Charge Storage Mechanism of Zn//4S6Q

**Fig. 5:**
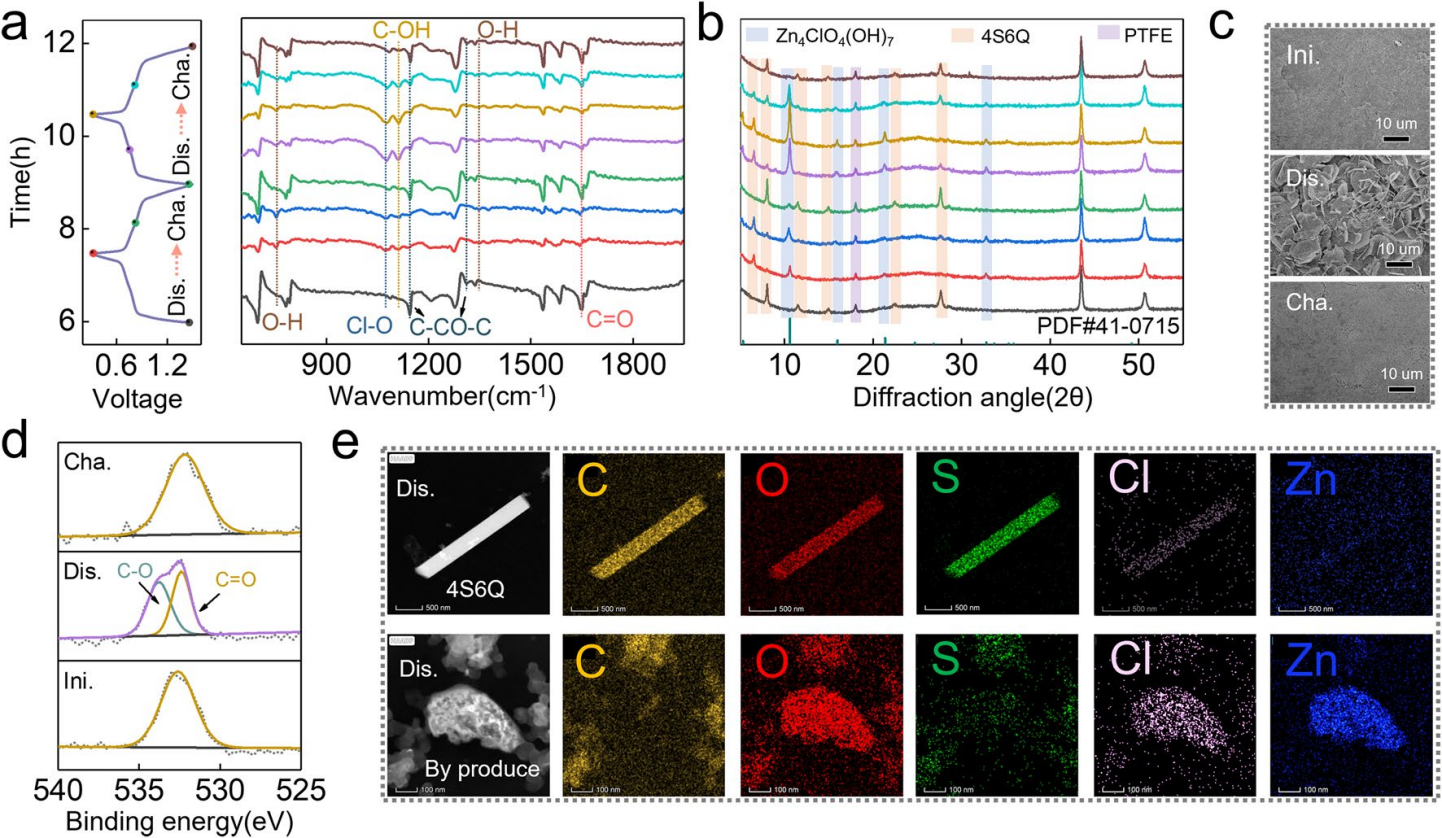
The charge storage mechanism of Zn//4S6Q. **a** *Ex situ* FTIR of 4S6Q electrodes at different states. **b** *Ex situ* XRD patterns of 4S6Q electrodes at different states. **c** *Ex situ* SEM images of 4S6Q electrodes at different states. **d** *Ex situ* XPS of O 1*s*. **e** TEM-mapping images of 4S6Q material and by-product at discharged state

The ion storage mechanism of 4S6Q material is studied by ex situ FTIR. As shown in Fig. 5a, the vibration peaks of the C=O group (1650 cm^−1^) and C–CO–C (1143 and 1310 cm^−1^) skeletons in quinone rings gradually weaken during the discharged process. Meanwhile, a new peak at 1110 cm^−1^ can be attributed to the signals of C–OH. The appearance of C–OH peak confirms the H^+^-involved reaction mechanism. Moreover, a new peak at 1076 cm^−1^, which the corresponds to the stretching vibration peaks of Cl–O, appears at discharged state [47]. Commonly, the H^+^ in electrolyte can participate in electrode reactions, resulting in interfacial pH imbalance and forming basic zinc salt by-products [48, 49]. Thus, the Cl–O group originates from the by-product of Zn_4_ClO_4_(OH)_7_, which further confirms the H^+^ uptake for 4S6Q during the discharged process. The O–H peaks of in-plane and out-of-plane deformation vibration are discovered at 750 and 1375 cm^−1^, which may come from the by-product or hydroquinone unit. These peaks can recover to the initial state after being charged to 1.5 V and show good reversibility in the following cycle. The ex situ XRD patterns of 4S6Q electrodes at different states are collected and shown in Fig. 5b. Obviously, these new diffraction peaks at 5.2, 10.6, 15.8, 21.3, and 32.8° are detected at the discharged state. These peaks match well with the standard Zn_4_ClO_4_(OH)_7_ (PDF#41–0715) and gradually disappear during the charged process, which reveals the H^+^-involved reaction mechanism for 4S6Q. In addition, the diffraction peaks of 4S6Q material at 8.0, 11.5, 14.9, 22.4, and 27.6° fade away during the discharged process, and a new diffraction peak at 6.5° appears. This result suggests that the space-stacked structure of 4S6Q is changed when inserted ions, which is further demonstrated by DFT calculations. As shown in Fig. S24, the optimized configurations of 4S6Q binding with one and two H^+^ ions show a plane structure. While they cannot maintain a plane structure when further increasing the number of H^+^, which may alter the space-stacked structure of 4S6Q. Benefiting from the –S– bonds, the flexible structure of 4S6Q makes it has a high tolerance for ions insertion. Thus, the 4S6Q shows good cycling stability. From the ex situ XRD patterns, the 4S6Q material displays good reversibility, verifying its excellent flexible structure. As shown in Fig. 5c, many flake pieces are discovered on the surface of the 4S6Q electrode at discharged state, corresponding to the morphology of Zn_4_ClO_4_(OH)_7_ by-product. These flake pieces disappear at charged state, revealing a high reversibility of H^+^ uptake/removal. The* ex situ* X-ray photoelectron spectroscopy (XPS) of O 1*s* is collected to verify the reaction sites of the 4S6Q compound. As shown in Fig. 5d, a peak is fitted in the initial state, corresponding to the O 1*s* in the C=O group. When discharged to 0.3 V, the peak of O 1*s* shifts to low binding energy. This peak is fitted to two components, corresponding to different chemical environments of O 1*s* in the C=O and C–O groups. When charged to 1.5 V, the C–O peak is absent, and the peak site shifts to initial state. The appearance of C–O is attributed to the transformation from quinone to hydroquinone.

As above discussions, the H^+^ as a carrier ion will participate in the electrode reaction for 4S6Q. However, it cannot be ignored many Zn^2+^ ions in 3.5 M Zn(ClO_4_)_2_ electrolyte. Whether the Zn^2+^ ions take part in the redox reaction of the 4S6Q electrode needs to be further clarified. Firstly, the TEM-mapping images of the 4S6Q electrode at discharge state are performed. If the Zn element is detected on the 4S6Q material, it may come from a by-product (Zn_4_ClO_4_(OH)_7_) or an insertion of Zn^2+^. As shown in Fig. 5e, the 4S6Q still maintains a rod-like morphology. The C, O, and S elements are obvious on the rod, and Cl and Zn elements are barely detected. Meanwhile, no signals of Cl and Zn elements are detected from the element linear scan image in Fig. S25, which is consistent with the EDS results (Table S2). These results suggest that the Zn^2+^ ions do not join in the reaction of 4S6Q. Thus, the discharge capacity of the Zn//4S6Q battery is contributed by H^+^ uptake. Further, the cation is altered to study the electrochemical behavior of the 4S6Q electrode. Here, the Zn//4S6Q battery based on 3.5 M Mg(ClO_4_)_2_ + 0.01 M Zn(ClO_4_)_2_ electrolyte is fabricated. The addition of a very small amount of zinc ions can maintain the high reversibility of the Zn anode. Commonly, different carrier ions will affect the redox peak shapes and potentials of electrode material. As shown in Fig. S26, the Zn//4S6Q battery based on 3.5 M Mg(ClO_4_)_2_ + 0.01 M Zn(ClO_4_)_2_ electrolyte shows two pairs of redox peaks, which are similar to that based on 3.5 M Zn(ClO_4_)_2_ electrolyte. This result implies the reaction mechanism of 4S6Q does not depend on the types of metal cation. Besides the metal cation, only the H^+^ can act as a carrier ion to bind with 4S6Q in 3.5 M Zn(ClO_4_)_2_ electrolyte. The Zn//4S6Q battery based on 3.5 M Mg(ClO_4_)_2_ + 0.01 M Zn(ClO_4_)_2_ electrolyte shows lower redox potential than that based on 3.5 M Zn(ClO_4_)_2_ electrolyte, which is caused by higher pH value of 3.5 M Mg(ClO_4_)_2_ + 0.01 M Zn(ClO_4_)_2_ electrolyte (pH = 4.02) than 3.5 M Zn(ClO_4_)_2_ electrolyte (pH = 1.53). Of note, the peak positions of the CV curve at 3.5 M Mg(ClO_4_)_2_ + 0.01 M Zn(ClO_4_)_2_ electrolyte can just coincide with that at 3.5 M Zn(ClO_4_)_2_ electrolyte after shifting (to eliminate the effect of pH), indicating the reaction mechanism of 4S6Q in different electrolytes is same. In addition, discharge platforms of Zn//4S6Q batteries at different electrolytes are almost coincide after shifting the discharge curve of Zn//4S6Q battery at 3.5 M Mg(ClO_4_)_2_ + 0.01 M Zn(ClO_4_)_2_ electrolyte (Fig. S27). Meanwhile, the Zn//4S6Q battery at 3.5 M Mg(ClO_4_)_2_ + 0.01 M Zn(ClO_4_)_2_ electrolyte also obtains a high discharge capacity of 205.4 mAh g^−1^. These results demonstrate that not Zn^2+^ but H^+^ can participate in the electrochemical reaction of 4S6Q in 3.5 M Zn(ClO_4_)_2_ electrolyte, which is also one of the reasons for the excellent rate performance of Zn//4S6Q battery.

### Low-Temperature Electrochemical Performance of Zn//4S6Q

**Fig. 6:**
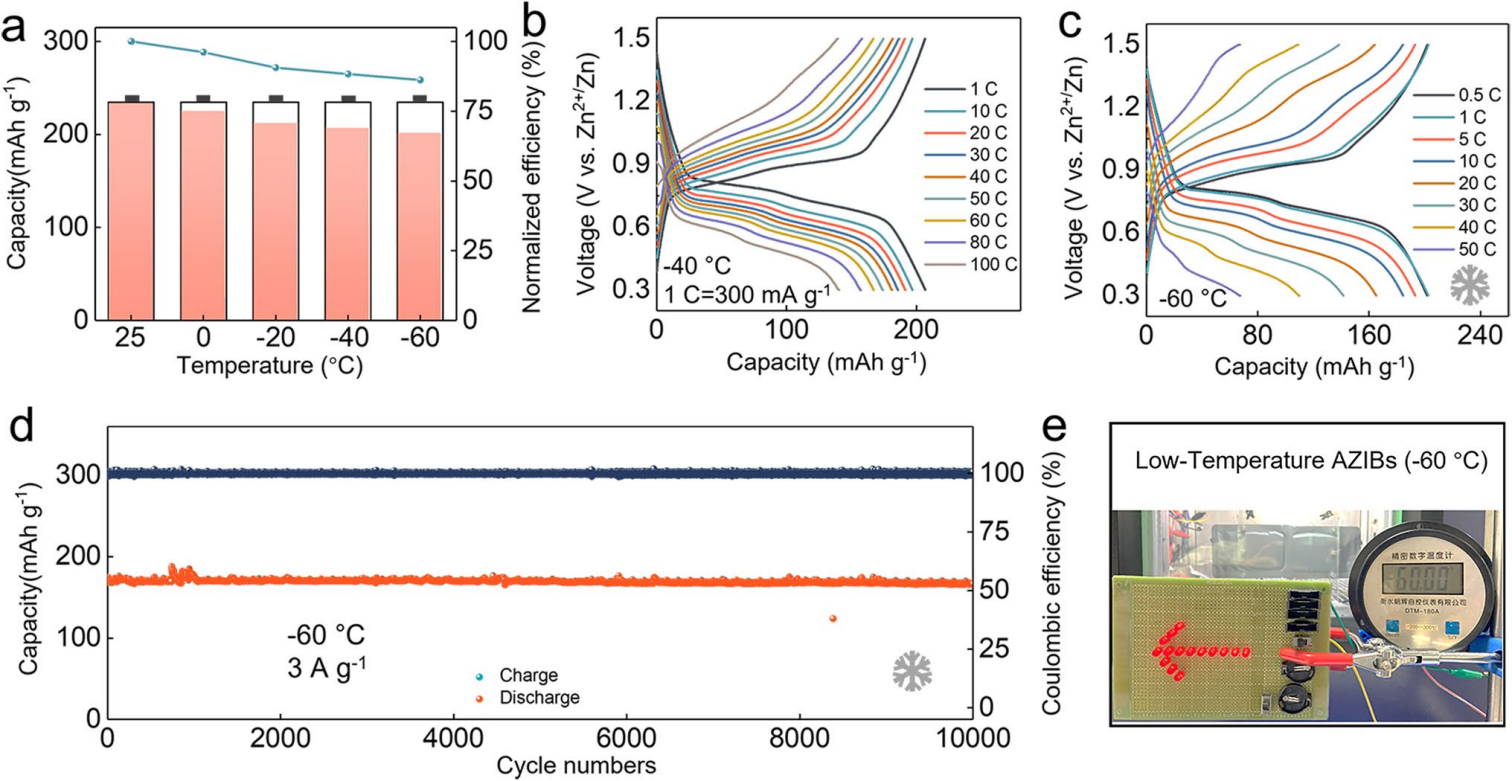
The low-temperature electrochemical performance of Zn//4S6Q. **a** Discharge capacity of Zn//4S6Q battery at different temperatures (at 150 mA g^−1^). **b** GCD curves of Zn//4S6Q battery at different current densities under − 40 °C. **c** GCD curves of Zn//4S6Q battery at different current densities under − 60 °C (1 C = 300 mA g^−1^). **d** Cycling stability of Zn//4S6Q battery at 3 A g^−1^ under − 60 °C. **e** Optical image of LED powered by two soft-packaged batteries in series at -60 °C

The low-temperature electrochemical performances of the Zn//4S6Q battery are tested due to the ultralow freezing point of 3.5 M Zn(ClO_4_)_2_ electrolyte (− 118 °C). As shown in Fig. 6a, the battery can normally operate and obtain a high discharge capacity of 201.7 mAh g^−1^ at − 60 °C, which is 86.2% of the discharge capacity at 25 °C. This system also exhibits remarkable rate capability at − 40 °C (Fig. 6b), which can be assigned to the low charge transfer impedances of 4S6Q at low temperature (Fig. S28). It obtains a discharge capacity of 140.4 mAh g^−1^ at 30 A g^−1^. When the temperature further reduces to − 60 °C, the discharge capacity still maintains 67.6 mAh g^−1^ of discharge capacity at 15 A g^−1^ (Fig. 6c). The battery can stably work at − 40 °C and keeps a discharge capacity of 205.1 mAh g^−1^ at 3 A g^−1^ after 3,000 cycles (Fig. S29). In particular, it has no capacity fading after 10,000 cycles at − 60 °C (Fig. 6d). The impressive low-temperature performance of Zn//4S6Q battery is profited by high conductivity and fast reaction kinetics of 4S6Q material. To verify its potential application, pouch cells are assembled and tested at − 60 °C. As shown in Fig. 6e, two pouch cells in series can drive the light-emitting diode (LED) light to work normally. The satisfactory low-temperature performances of the Zn//4S6Q battery make it show great promise for practical application in extremely cold conditions (Table S3).

## Conclusions

Summarily, novel small organic materials, namely 4S6Q and 4S4Q, are successfully synthesized by the molecule structure design method. The introduction of conjugated thioether (–S–) bonds not only extends the π-conjugated plane of whole molecules but also improves their conductivity. More importantly, the flexible molecular skeleton of 4S6Q and 4S4Q endows them with a robust tolerance for ions uptake/removal. Thus, the 4S6Q and 4S4Q show low solubleness and good electrochemical stability. As a result, the Zn//4S6Q battery based on the 3.5 M Zn(ClO_4_)_2_ electrolyte exhibits a high discharge capacity of 240 mAh g^−1^ at 150 mA g^−1^ and superior cycling life with no capacity fading after 20,000 cycles at 3 A g^−1^. 208.6 mAh g^−1^ of discharge capacity is achieved even at a high current density of 30 A g^−1^. Comprehensive characterizations identify the robust redox activity of carbonyl groups and reveal the H^+^-storage mechanism for 4S6Q material. Impressively, this battery can stably operate at − 60 °C and has no capacity reduction after 10,000 cycles at 3 A g^−1^. This work proves a route to design robust small organic material and extends the application of AZIBs at low-temperature conditions.

### Supplementary Information

Below is the link to the electronic supplementary material.

Supplementary file1 (PDF 1860 KB)
