# Supplementary material for: Molecular Engineering Design for High-Performance Aqueous Zinc-Organic Battery
Source: Nanomicro Lett. 2023 Jan 13;15:36. doi: 10.1007/s40820-022-01009-x (PMC9839927; doi:10.1007/s40820-022-01009-x)
Supplement: Supplementary file 1 — Supplementary file1 (PDF 1860 KB) [file 40820_2022_1009_MOESM1_ESM.pdf]

Supporting Information for

Molecular Engineering Design for High-Performance Aqueous Zinc-Organic Battery

Tianjiang Sun[1], Weijia Zhang[1], Qingshun Nian[2], Zhanliang Tao[1, *]

[1]Key Laboratory of Advanced Energy Materials Chemistry (Ministry of Education), Renewable Energy Conversion and Storage Center, College of Chemistry, Nankai University, Haihe Laboratory of Sustainable Chemical Transformations, Tianjin 300071, P. R. China

[2]Hefei National Laboratory for Physical Science at the Microscale, CAS Key Laboratory of Materials for Energy Conversion, Department of Materials Science and Engineering, University of Science and Technology of China Hefei, Anhui 230026, P. R. China

*Corresponding author. E-mail: taozhl@nankai.edu.cn (Zhanliang Tao)

Supplementary Figures and Tables

S

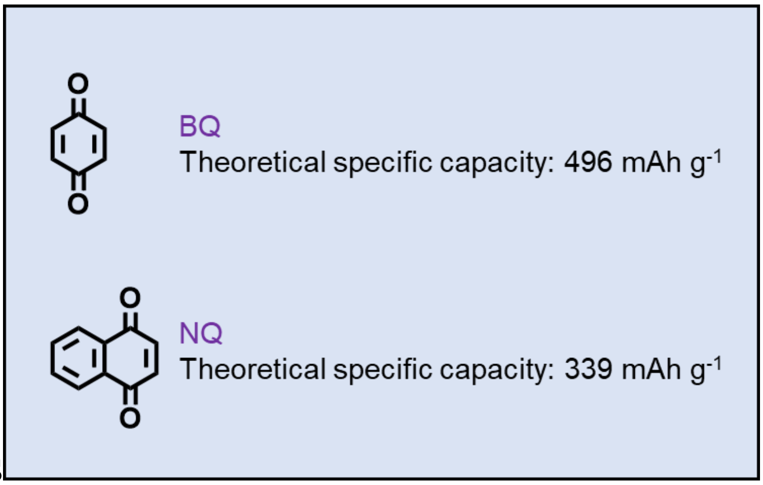

Fig. S1 Molecular structures of BQ and NQ compounds

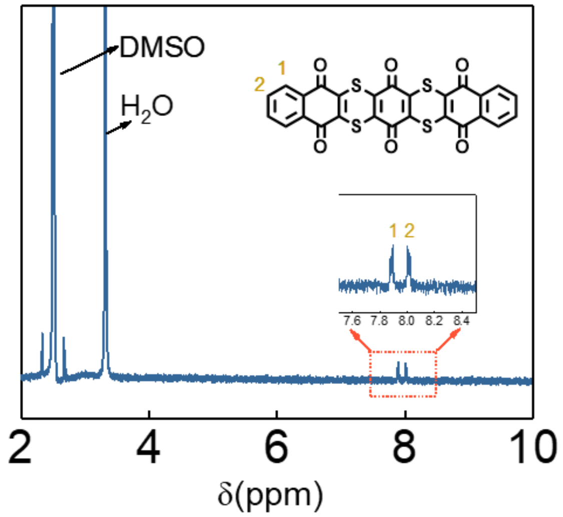

Fig. S2 ^{1}H NMR spectra of 4S6Q

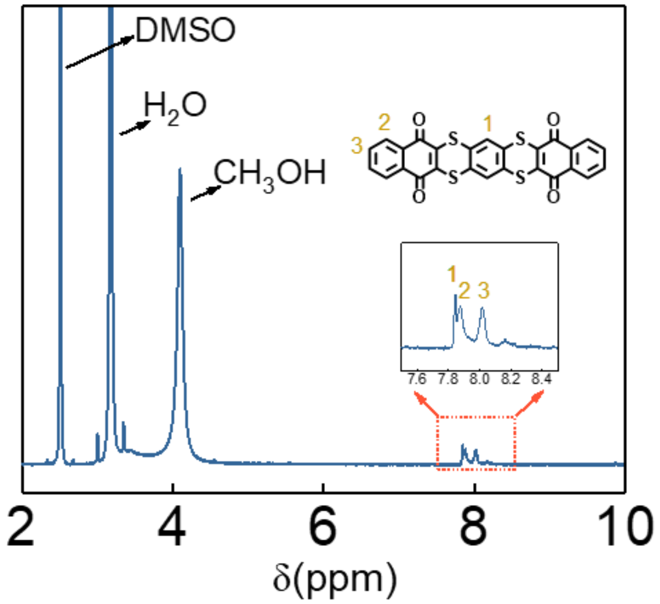

Fig. S3 ^{1}H NMR spectra of 4S4Q

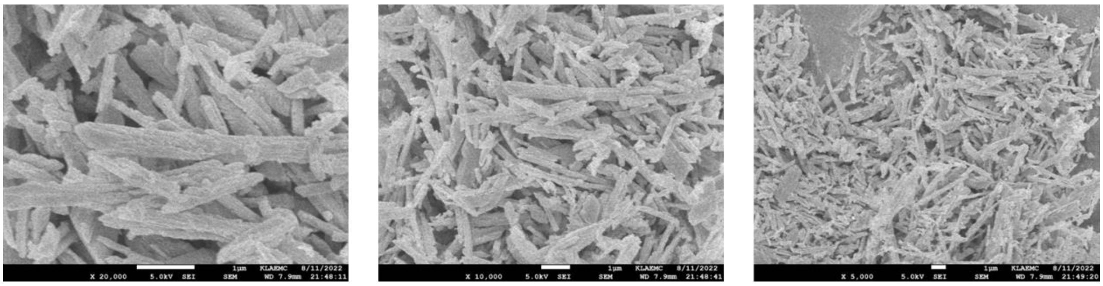

Fig. S4 SEM images of 4S6Q

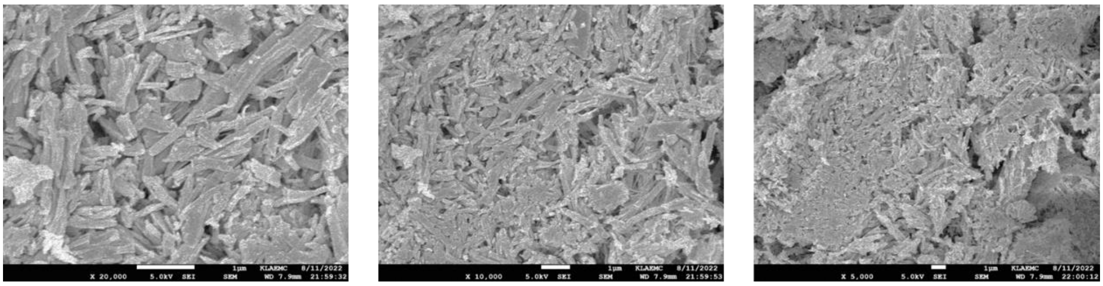

Fig. S5 SEM images of 4S4Q

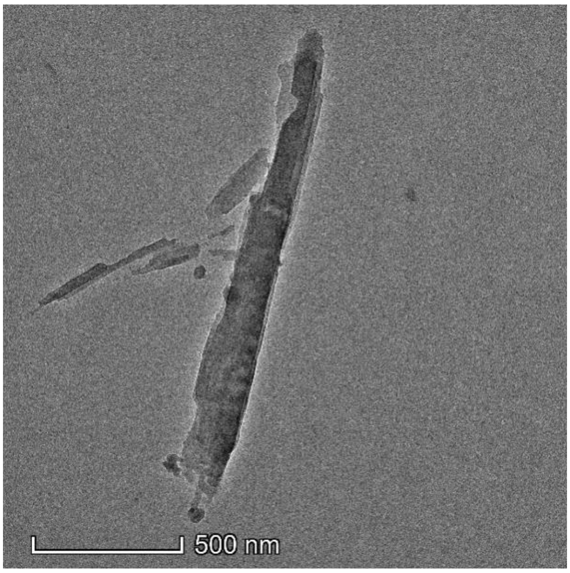

Fig. S6 TEM image of 4S4Q

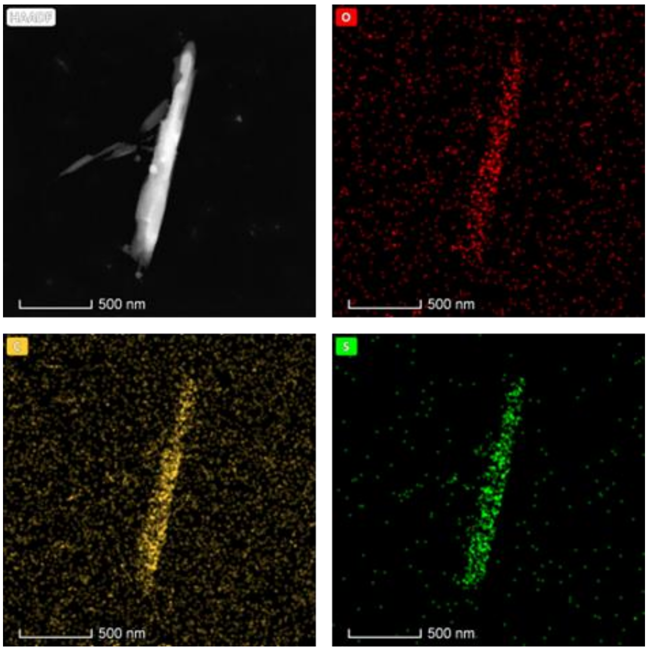

Fig. S7 TEM-Mapping images of 4S4Q

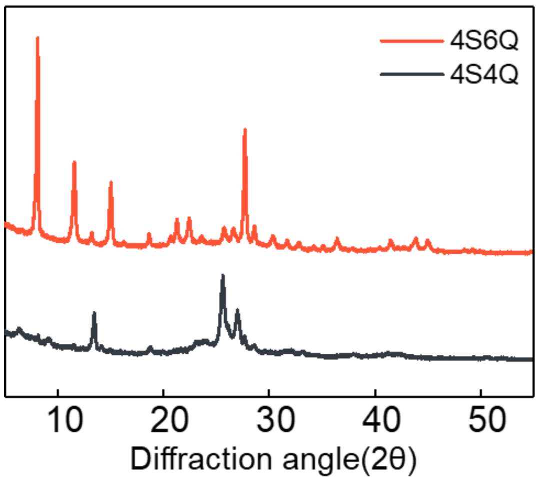

Fig. S8 XRD patterns of 4S6Q and 4S4Q

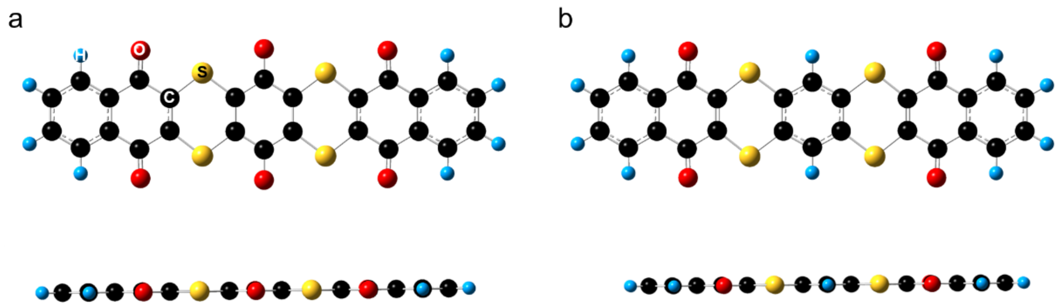

Fig. S9 Optimized structures of (**a**) 4S6Q; and (**b**) 4S4Q

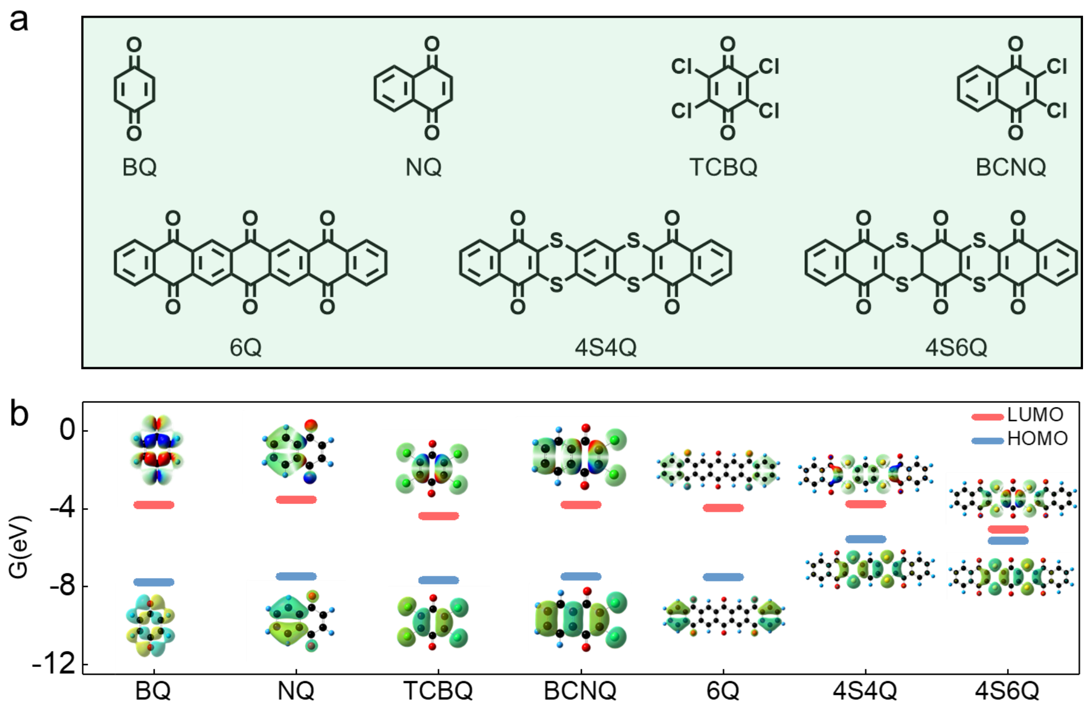

Fig. S10 Energy levels of LUMO and HOMO for different compounds

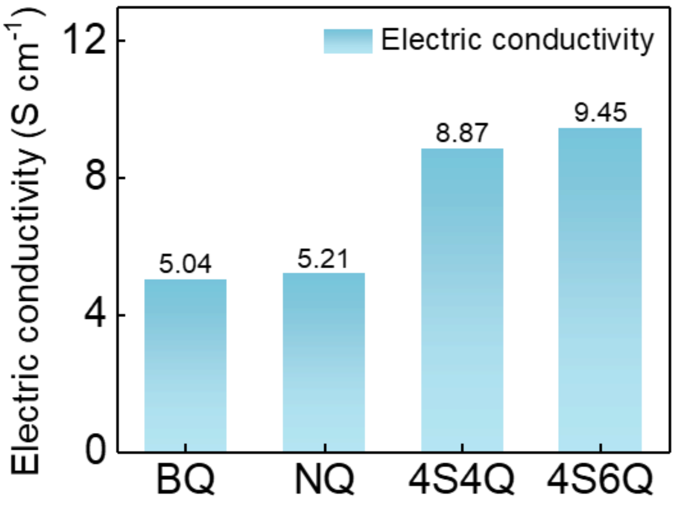

Fig. S11 Electric conductivities of different compounds

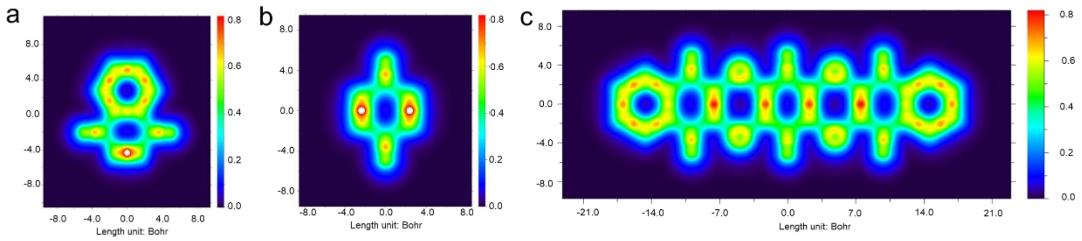

Fig. S12 LOL-π image of a) NQ; b) BQ; c) 4S6Q

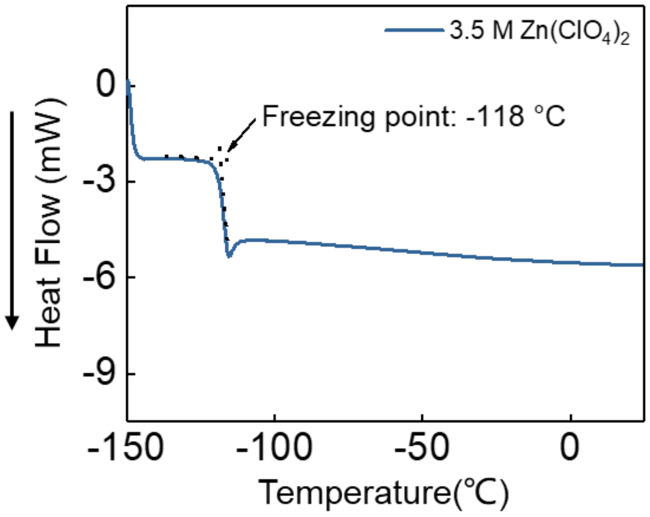

Fig. S13 Freezing point of 3.5 M Zn(ClO$_4$)$_2$ electrolyte

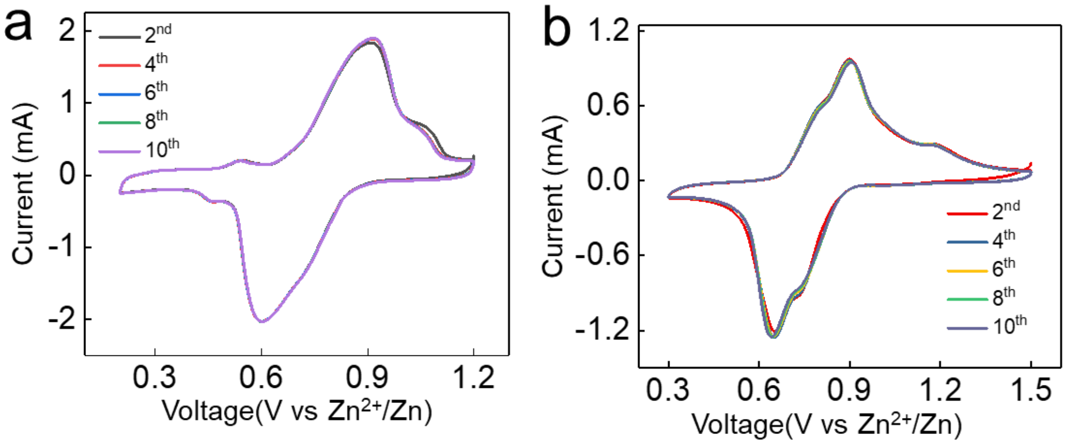

Fig. S14 CV curves of **a**) Zn//4S4Q battery at 5 mV s^{-1}; **b**) Zn//4S6Q battery at 5 mV s^{-1}

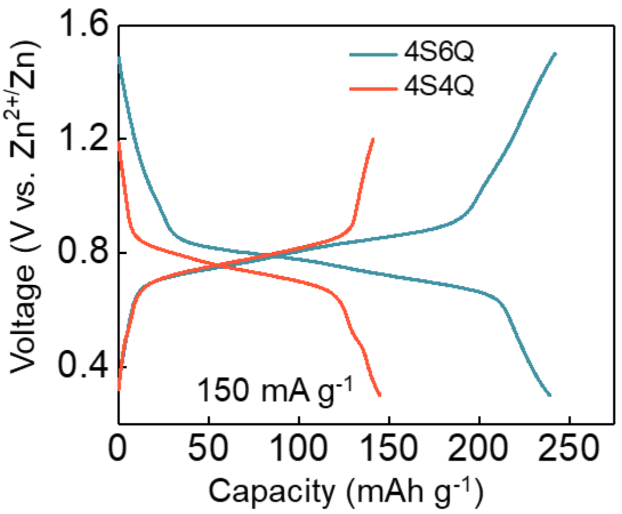

Fig. S15 GCD curves of Zn//4S4Q and Zn//4S6Q battery at 150 mA g^{-1}

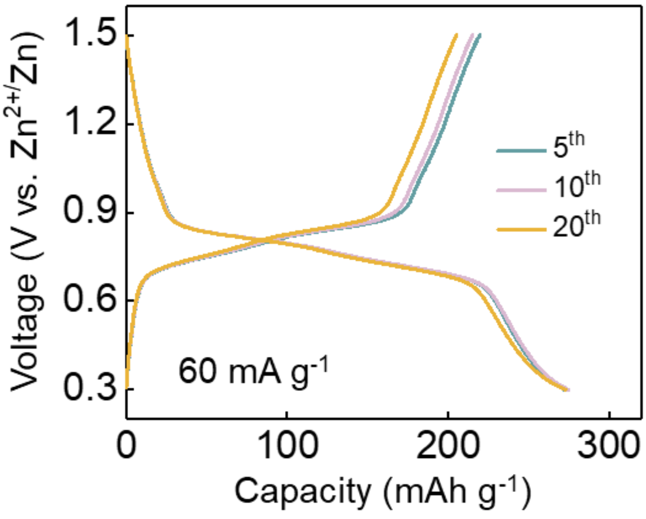

Fig. S16 GCD curves of the Zn//4S6Q battery at 60 mA g^{-1}

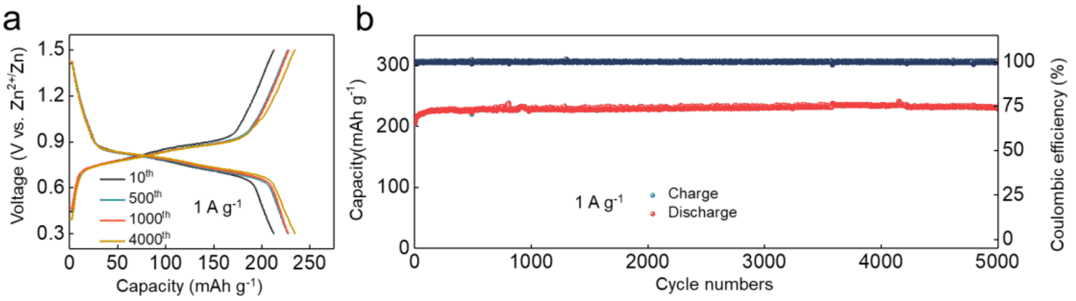

Fig. S17 a) GCD curves of Zn//4S6Qat 1 A g^{-1}. **b**) Cycling stability of Zn//4S6Q battery at 1 A g^{-1}

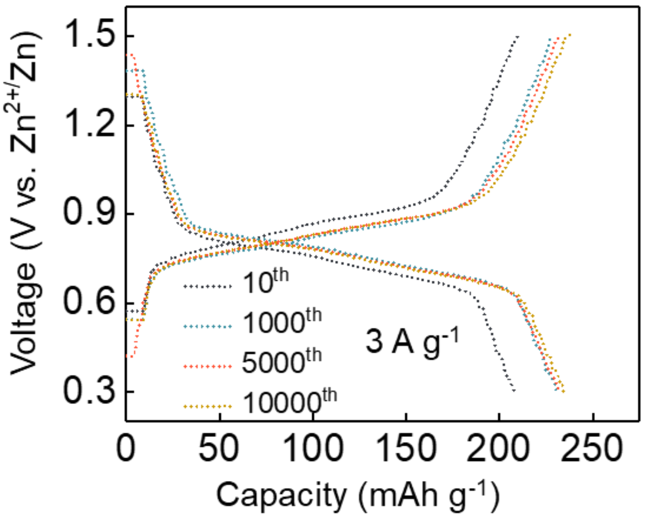

Fig. S18 GCD curves of Zn//4S6Q battery at 3 A g^{-1}

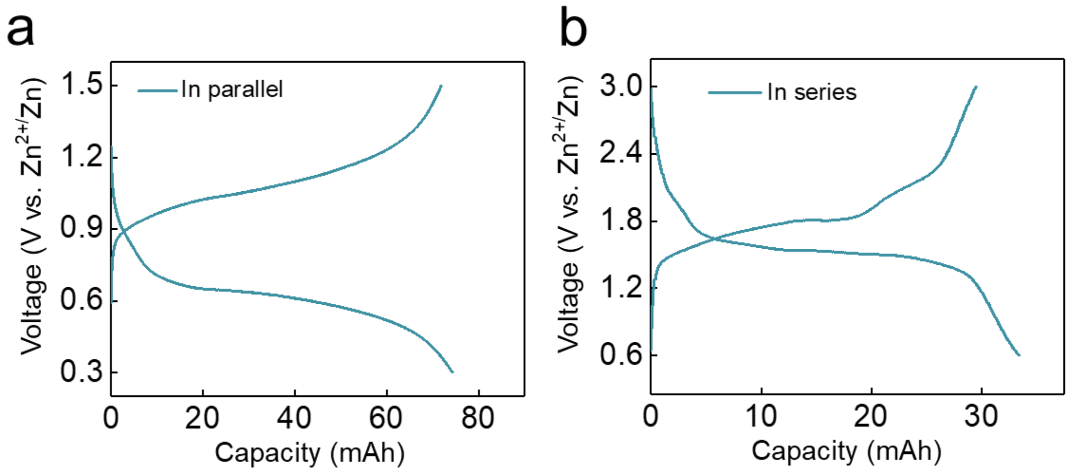

Fig. S19 Pouch cells are connected in **a**) parallel; **b**) series

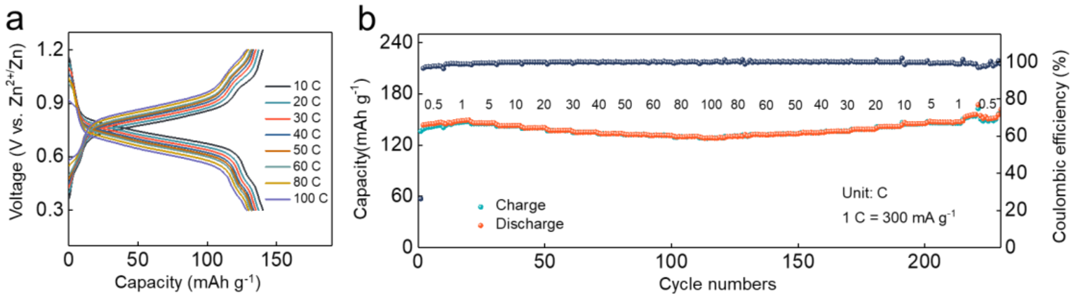

Fig. S20 a) GCD curves of Zn//4S4Q battery at different current densities. **b**) Rate capability of Zn//4S4Q battery

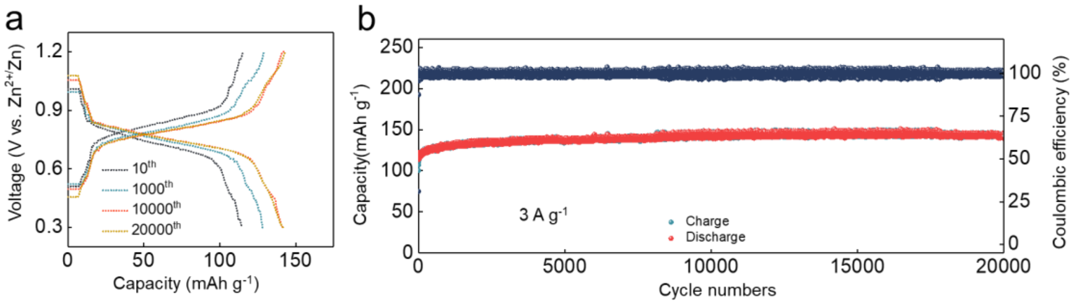

Fig. S21 a) GCD curves of Zn//4S4Q battery at 3 A g^{-1}. **b**) Cycling stability of Zn//4S4Q battery at 3 A g^{-1}

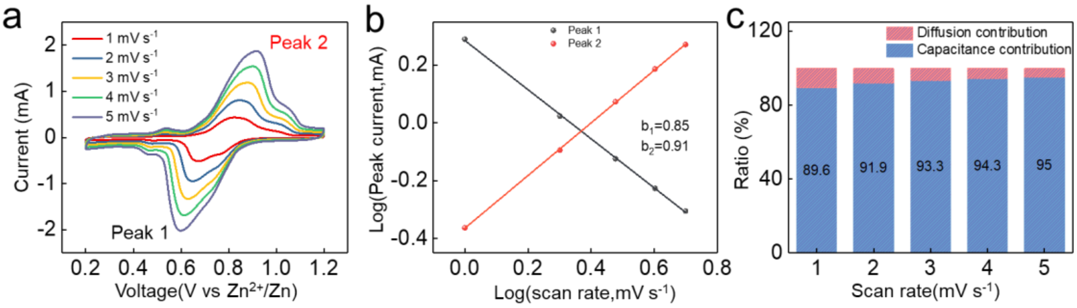

Fig. S22 a) CV curves of Zn//4S4Q battery at different scan rates. **b**) Calculated *b* values. **c**) Ratios of capacitance and diffusion contribution at different scan rates

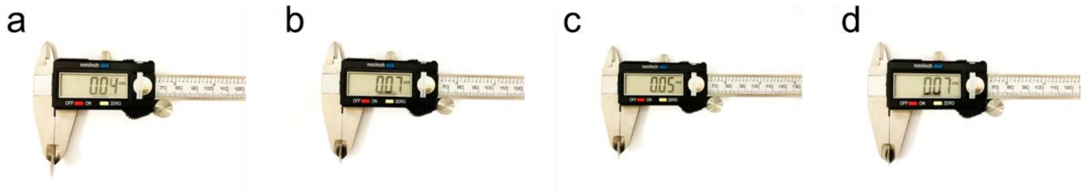

Fig. S23 a) Thickness of the current collector for the 4S4Q electrode. **b**) Thickness of the whole 4S4Q electrode. **c**) Thickness of the current collector for the 4S6Q electrode. **d**) Thickness of the whole 4S6Q electrode

The following equation can calculate the ionic conductivity:

$$\sigma = \frac{L}{R_{ct}S}$$

where σ refers to the ionic conductivity, L refers to the thickness of active material, R_{ct} corresponds to the charge transfer impedance (124.9 Ω for 4S4Q and 33.8 Ω for 4S6Q), S corresponds to the area of active material (the diameter of active material is 8 mm). Thus, the ionic conductivities of 4S4Q and 4S6Q are calculated to be 4.78 and 11.76 mS cm^{-1}, respectively.

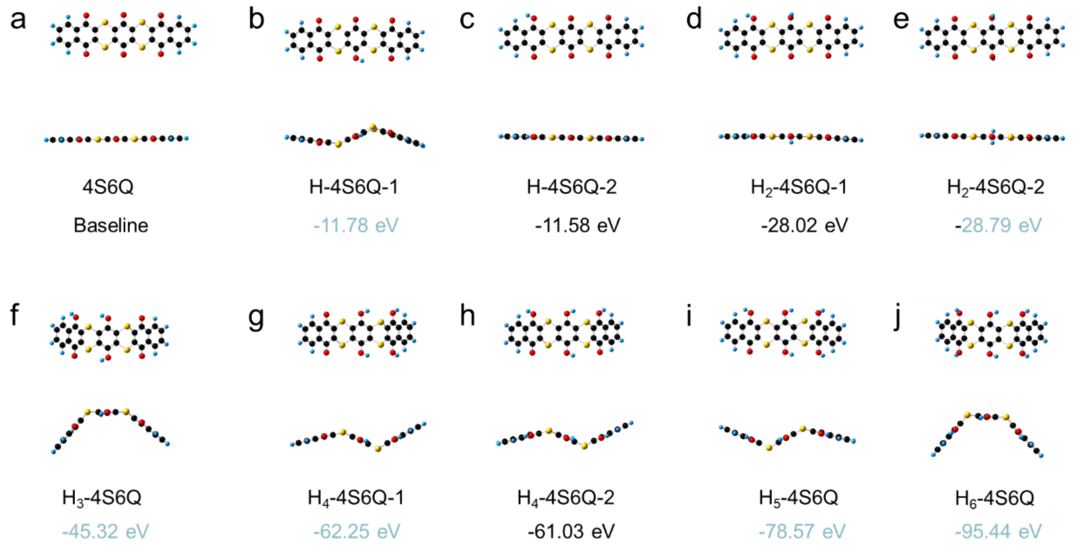

Fig. S24 The optimized structure and energy of **a**) 4S6Q; **b**) H-4S6Q-1; **c**) H-4S6Q-2; **d**) H_2-4S6Q-1; **e**) H_2-4S6Q-2; **f**) H_3-4S6Q; **g**) H_4-4S6Q-1; **h**) H_4-4S6Q-2; **i**) H_5-4S6Q; **j**) H_6-4S6Q

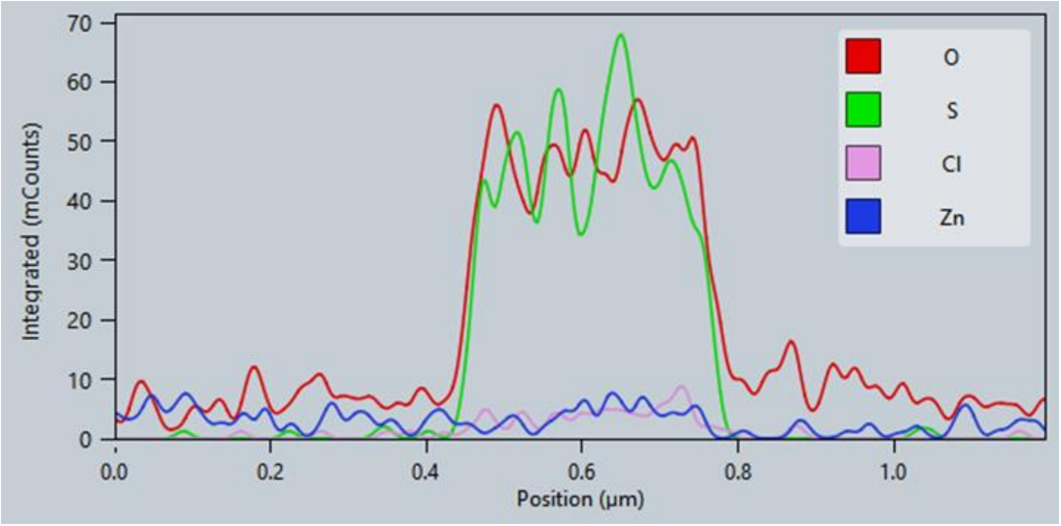

Fig. S25 Element linear scan image of 4S6Q electrode at discharged state

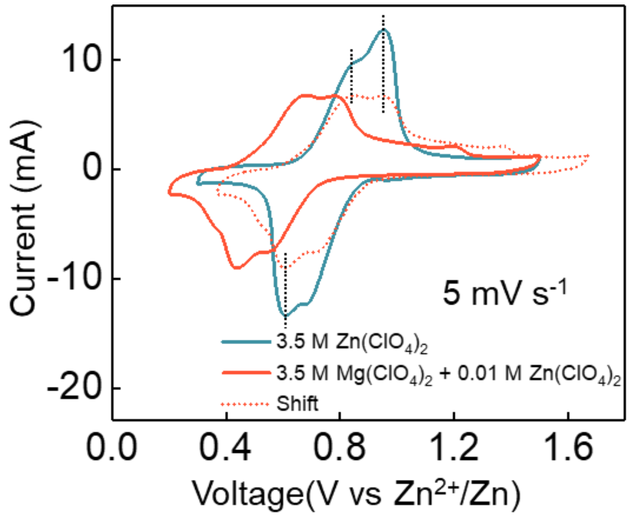

Fig. S26 CV curves of Zn//4S6Q battery at different electrolytes

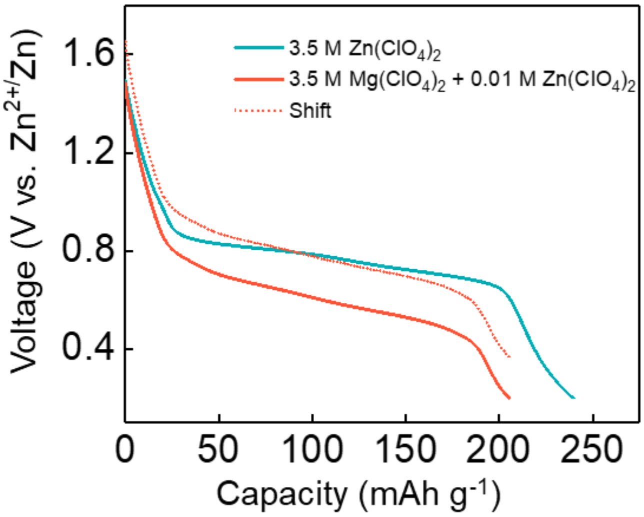

Fig. S27 GCD curves of Zn//4S6Q battery at different electrolytes

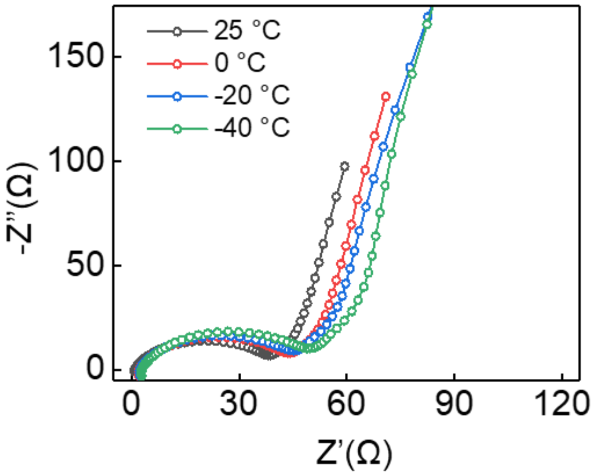

Fig. S28 Impedances of 4S6Q at low-temperature conditions

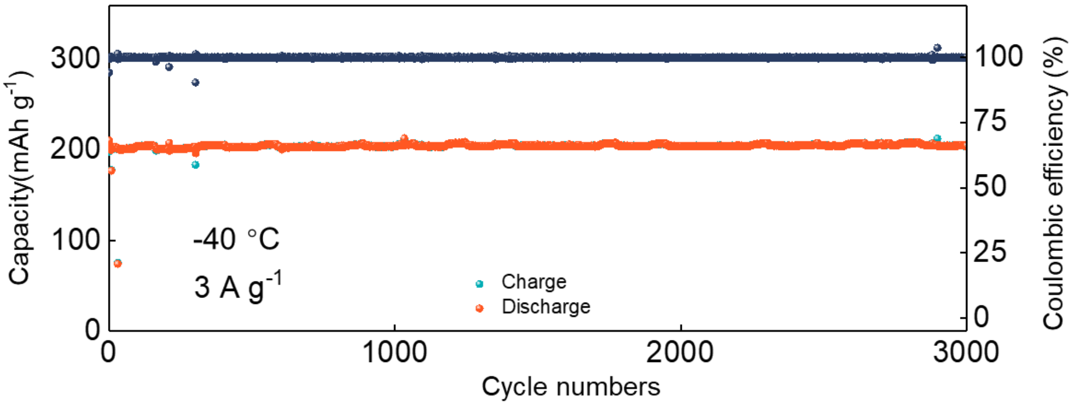

Fig. S29 Cycle stability of Zn//4S6Q battery at -40 °C

Table S1 Comparison of electrochemical performance for various of organic materials

Molecular structure	Discharge capacity (mAh g^{-1})/current density (A g^{-1})	Cycle number/ current density (A g^{-1})/capacity retention (%)	Refs.
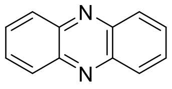	232/0.02<br>80/1	100/0.1/89<br>1000/1/79	[S1]
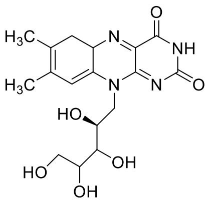	113.5/0.03<br>95.8/5	100/0.1/66.1<br>3000/5/92.7	[S2]
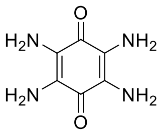	303/0.01<br>213/5	1000/5/83.3	[S3]
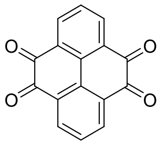	336/0.04<br>162/5	1000/3/70	[S4]
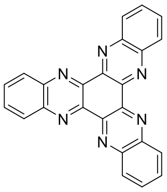	405/0.1<br>123/20	5000/5/93.3	[S5]

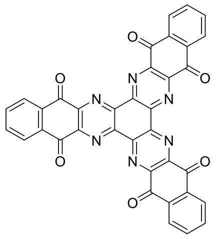	482.5/0.2<br>177.5/9	11000/5/99.3	[S6]
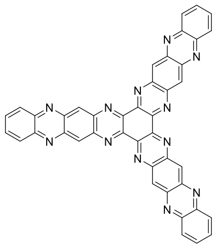	257/5<br>144/100	30000/30/92.7	[S7]
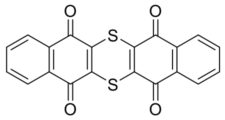	210.9/0.05<br>97/2	150/0.1/94%<br>23000/2/83.8%	[S8]
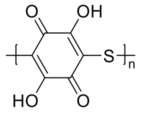	215/0.05<br>161/5	1000/1/no capacity fading	[S9]
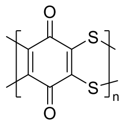	205/0.05<br>176/10	10000/20/75.2%	[S10]
PANI	191/0.05<br>95/5	3000/5/92	[S11]
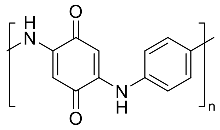	329/0.1<br>277/20	4800/1/85	[S12]
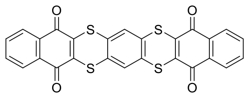	145.8/0.15<br>128.3/30	20000/3/ no capacity fading	This work
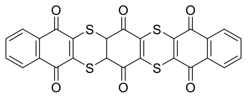	240/0.15<br>208/30	500/0.15/ no capacity fading<br>20000/3/ no capacity fading	

Table S2 EDS of 4S6Q electrode at discharged state

Element	Atomic Fraction (%)	Mass Fraction (%)
O	69.12	52.68
S	30.75	46.97
Cl	0.03	0.05
Zn	0.10	0.30

Table S3 The comparison of electrochemical performance for various of organic materials at low-temperature conditions

Cathode	Electrolyte	Operated temperature	Discharge capacity (mAh g^{-1})/current density (A g^{-1})	Cycle number/ current density (A g^{-1})/capacity retention (%)	Refs.
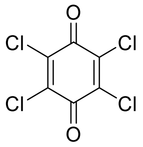	4 M $Zn(BF_4)_2$	-60 °C	86.1/0.022	50/0.022/97.3	[S13]
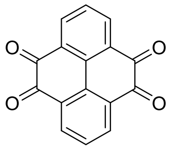	3.5 M $Mg(ClO_4)_2$ + 1 M $Zn(ClO_4)_2$	-70 °C	101/0.2<br>71/1.2	100/0.2/ no capacity fading	[S14]
PANI	7.5 M $ZnCl_2$	-70 °C	106.2/0.02<br>8/5	2000/0.2/ no capacity fading	[S15]
Activated carbon	3 M $Zn(ClO_4)_2$	-60 °C	45.88/0.5	70000/1/ no capacity fading	[S16]
Amorphous V_2O_5	2 M $Zn(CF_3SO_3)_2$	-30 °C	285/0.1<br>194.1/1	1000/0.5/81.7	[S17]
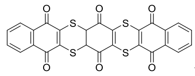	3.5 M $Zn(ClO_4)_2$	-60 °C	201.7/0.15<br>67.6/15	10000/3/ no capacity fading	This work

Supplementary References

[S1] W. Wang, V.S. Kale, Z. Cao, S. Kandambeth, W. Zhang et al., Phenanthroline covalent organic framework electrodes for high-performance zinc-ion supercapattery. ACS Energy Lett. **5**, 2256 (2020). https://doi.org/10.1021/acsenergylett.0c00903

[S2] L. Cheng, Y. Liang, Q. Zhu, D. Yu, M. Chen et al., Bio-inspired isoalloxazine redox moieties for rechargeable aqueous zinc-ion batteries. Chem. Asian J. **15**, 1290 (2020). https://doi.org/10.1002/asia.202000283

[S3] Z. Lin, H.Y. Shi, L. Lin, X. Yang, W. Wu et al., A high capacity small molecule quinone cathode for rechargeable aqueous zinc-organic batteries. Nat. Commun. **12**, 4424 (2021). https://doi.org/10.1038/s41467-021-24701-9

[S4] Z. Guo, Y. Ma, X. Dong, J. Huang, Y. Wang et al., An environmentally friendly and flexible aqueous zinc battery using an organic cathode. Angew. Chem. Int. Ed. **57**, 11737 (2018). https://doi.org/110.1002/anie.201807121

[S5] Z. Tie, L. Liu, S. Deng, D. Zhao, Z. Niu, Proton insertion chemistry of a zinc–organic battery. Angew. Chem. Int. Ed. **59**, 4920 (2020). https://doi.org/10.1002/anie.201916529

[S6] Y. Chen, J. Li, Q. Zhu, K. Fan, Y. Cao et al., Two-dimensional organic

supramolecule via hydrogen bonding and π–π stacking for ultrahigh capacity and long-life aqueous zinc–organic batteries. Angew. Chem. Int. Ed., e202116289 (2022). https://doi.org/10.1002/anie.202116289
[S7] S. Li, J. Shang, M. Li, M. Xu, F. Zeng et al., Design and Synthesis of a π-Conjugated N-Heteroaromatic Material for Aqueous Zinc–Organic Batteries with Ultrahigh Rate and Extremely Long Life. Adv. Mater., e2207115 (2022). https://10.1002/adma.202207115
[S8] Y. Wang, C. Wang, Z. Ni, Y. Gu, B. Wang et al., Binding zinc ions by carboxyl groups from adjacent molecules toward long-life aqueous zinc–organic batteries. Adv. Mater. **32**, e2000338 (2020). https://10.1002/adma.202000338
[S9] T. Sun, Z.J. Li, Y.F. Zhi, Y.J. Huang, H.J. Fan et al., Poly(2,5-dihydroxy-1,4-benzoquinonyl sulfide) as an efficient cathode for high-performance aqueous zinc–organic batteries. Adv. Funct. Mater. **31**, 2010049 (2021). https://10.1002/adfm.202010049
[S10] J. Xie, F. Yu, J. Zhao, W. Guo, H.-L. Zhang et al., An irreversible electrolyte anion-doping strategy toward a superior aqueous Zn-organic battery. Energy Storage Mater. **33**, 283 (2020). https://10.1016/j.ensm.2020.08.027
[S11] F. Wan, L. Zhang, X. Wang, S. Bi, Z. Niu et al., An aqueous rechargeable zinc-organic battery with hybrid mechanism. Adv. Funct. Mater. **28**, 1804975 (2018). https://10.1002/adfm.201804975
[S12] H. Zhang, D. Xu, L. Wang, Z. Ye, B. Chen et al., A polymer/graphene composite cathode with active carbonyls and secondary amine moieties for high-performance aqueous zn-organic batteries involving dual-ion mechanism. Small **17**, e2100902 (2021). https://10.1002/smll.202100902
[S13] T. Sun, X. Yuan, K. Wang, S. Zheng, J. Shi et al., An ultralow-temperature aqueous zinc-ion battery. J. Mater. Chem. A **9**, 7042 (2021). https://10.1039/d0ta12409e
[S14] T. Sun, S. Zheng, H. Du, Z. Tao, Synergistic effect of cation and anion for low-temperature aqueous zinc-ion battery. Nano-Micro Lett. **13**, 204 (2021). https://10.1007/s40820-021-00733-0
[S15] Q. Zhang, Y. Ma, Y. Lu, L. Li, F. Wan et al., Modulating electrolyte structure for ultralow temperature aqueous zinc batteries. Nat. Commun. **11**, 4463 (2020). https://10.1038/s41467-020-18284-0
[S16] Y. Sun, H. Ma, X. Zhang, B. Liu, L. Liu et al., Salty ice electrolyte with superior ionic conductivity towards low-temperature aqueous zinc ion hybrid capacitors. Adv. Funct. Mater. **31**, 2101277 (2021). https://10.1002/adfm.202101277
[S17] Q. Zhang, K. Xia, Y. Ma, Y. Lu, L. Li et al., Chaotropic anion and fast-kinetics cathode enabling low-temperature aqueous zn batteries. ACS Energy Lett. **6**, 2704 (2021). https://10.1021/acsenergylett.1c01054
